# Synthesis of warfarin analogs: conjugate addition reactions of alkenyl-substituted N-heterocycles with 4-hydroxycoumarin and related substrates†

**DOI:** 10.1039/d3ra00251a

**Published:** 2023-02-06

**Authors:** Benjamin Goka, Douglas A. Klumpp

**Affiliations:** a Department of Chemistry and Biochemistry, Northern Illinois University DeKalb Illinois 60115 USA dklumpp@niu.edu

## Abstract

We have developed a procedure for the Michael addition of 4-hydroxycoumarins to vinyl-substituted N-heterocycles. The chemistry is also suitable for thiocoumarins and quinolinones. A mechanism is proposed involving nucleophilic attack at the vinyl-group of the protonated N-heterocycle.

## Abstract 2

We have developed a procedure for the Michael addition of 4-hydroxycoumarins to vinyl-substituted N-heterocycles.

## Graphical abstract

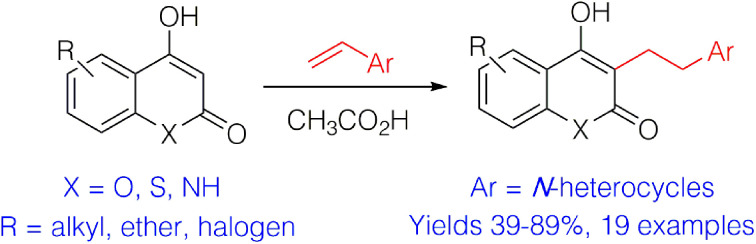

## Introduction

Warfarin (1) is a clinically important anticoagulant drug.^1^ It was first approved for use in the mid-1950s and warfarin is currently listed on the World Health Organization's List of Essential Medicines.^2^ The substance is commonly prepared using a base-catalyzed reaction of 4-hydroxycoumarin (2) with benzalacetone (eqn (1)).^3^ Enantioselective addition reactions have also been developed.^4^ Our group recently described the Michael addition reactions of 1,3-dicarbonyl compounds with vinyl-substituted N-heterocycles (eqn (2)).^5^ Based on this chemistry, we hypothesized that 4-hydroxycoumarins would exhibit similar nucleophilic reactivity with vinyl-substituted N-heterocycles. In the following Communication, we describe a convenient method for the synthesis of heterocycle-containing analogs of warfarin.1
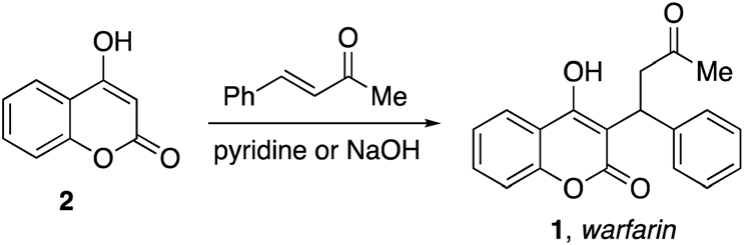
2
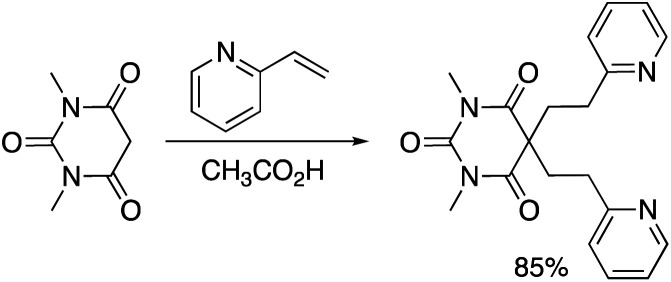


## Results and discussion

Using our previous methodology,^5^ 4-hydroxycoumarin was reacted with 2-vinylpyridine and acetic acid in acetonitrile and the addition product 3 was isolated in 69% yield (Table 1). The product was exceptionally difficult to purify using chromatography, so a methodology was developed with crystalizing the product directly from the crude product mixture. Similar addition products (3–8) were prepared from substituted 4-hydroxycoumarins, including halogen, alkyl, and the methoxy-substituted systems. A modest yield of product 9 was obtained from 5-nitro-2-vinylpyridine and 4-hydroxycoumarin. The conversions were also accomplished with 4-vinylpyridine, providing compounds 10–14 in fair to good yields. The lower yields seem be associated with systems that did not crystalize well from the crude product mixtures. For example, 4-hydroxycoumarin reacted with di-(4-pyridyl)ethylene but inefficient crystallization provided only a 39% isolated yield of compound 15. Additionally, we found some systems slowly formed products from double addition reactions. These minor biproducts were identified from mass spectral analysis and NMR analysis of crude product mixtures. The data suggests C- and O-alkylation products (*i.e.*16). To suppress formation of these biproducts, some of the conversions were best conducted with equimolar ratios of the vinylpyridine and 4-hydroxycoumarin.

**Table 1:** Products and yields from the reactions of 4-hydroxycoumarins with vinylpyridines

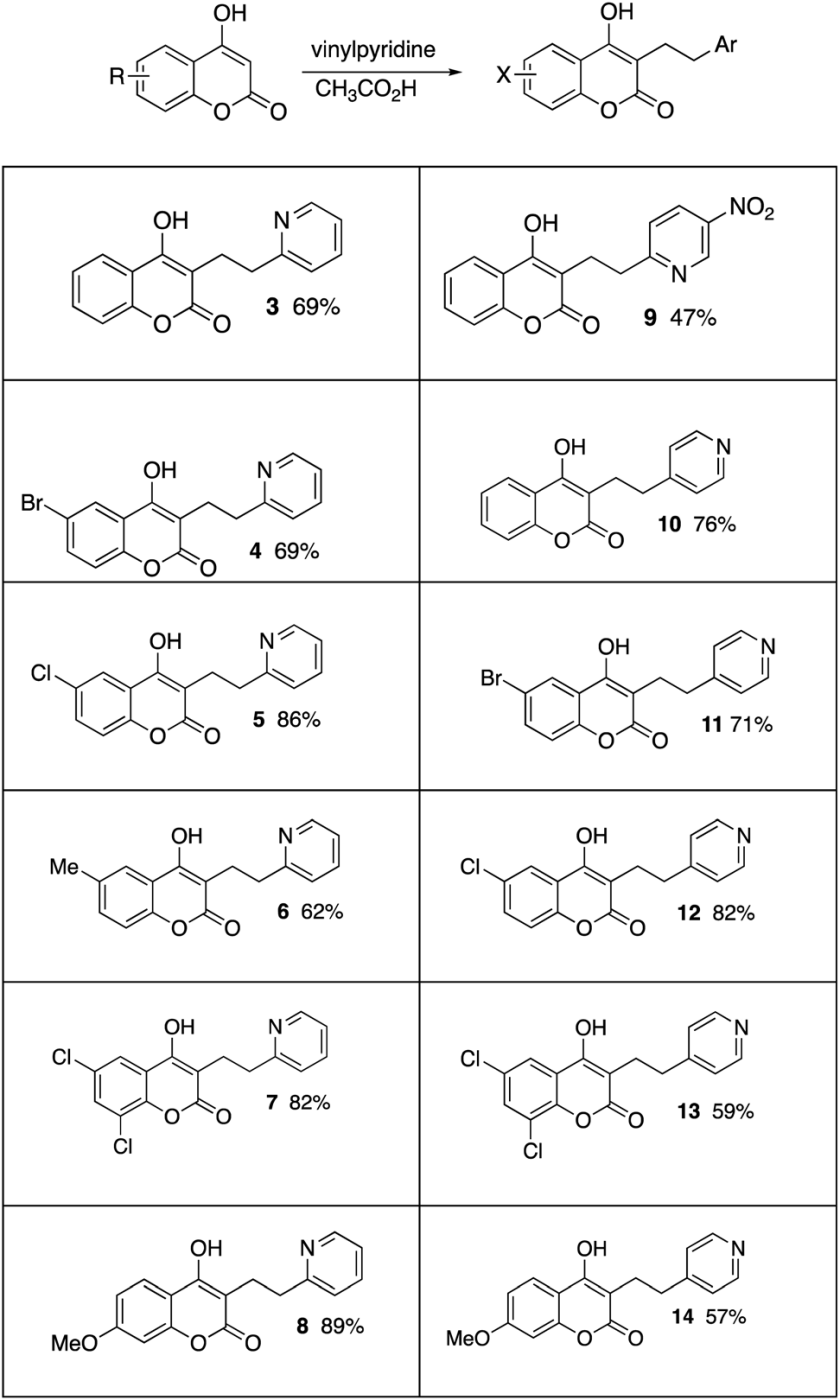

**Scheme 1:**
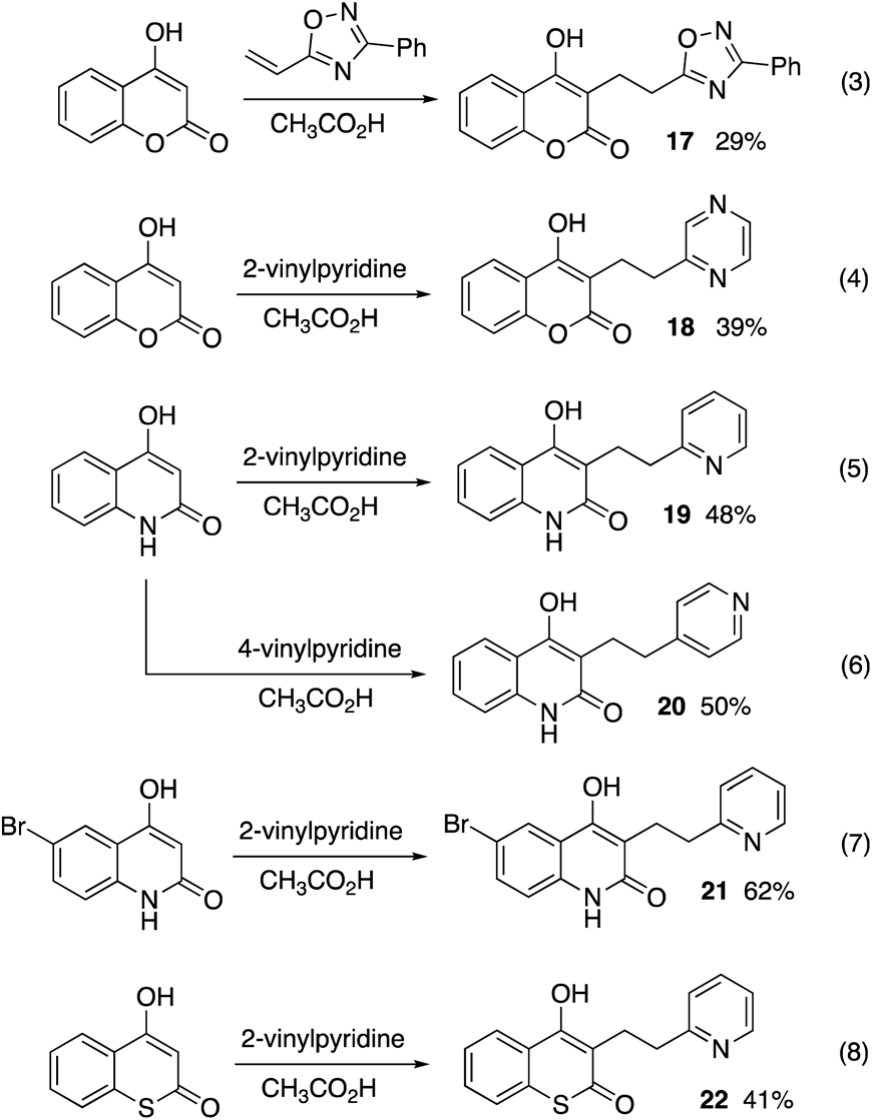
Addition reactions with varied heterocycles.

Over the past 50 years, several types of vinyl-substituted heterocycles have been shown to be reactive as Michael acceptors.^6^ We have found that 4-hydroxycoumarin also reacts with other types of olefinic heterocycles. When 4-hydroxycoumarin is reacted with a vinyl-substituted 1,2,4-oxadiazole, product 17 is obtained, albeit in low isolated yield (Scheme 1, eqn (3)). Likewise, vinylpyrazine gives the adduct 18 in 39% yield from a reaction with 4-hydroxycuomarin (eqn (4)). The chemistry is also compatible with closely related nucleophiles. Thus, 4-hydroxyquinolin-2(1*H*)-one reacts with 2- and 4-vinylpyridine to give products 19–20 in fair yields (eqn (5) and (6)). Similarly, the brominated 4-hydroxyquinolin-2(1*H*)-one gives compound 21 from 2-vinylpyridine (eqn (7)). The adduct (22) from 4-hydroxy-2*H*-thiochromen-2-one is also formed in fair yield from 2-vinylpyrdine (eqn (8)).
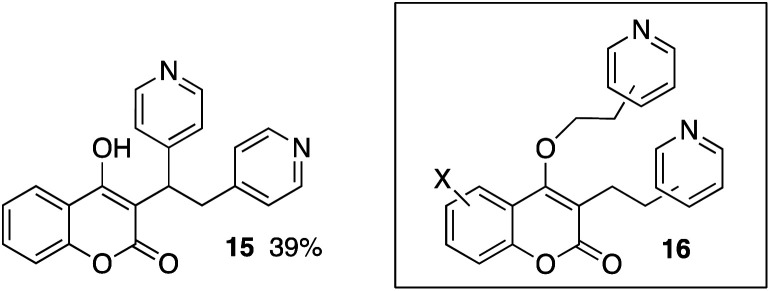


**Scheme 2:**
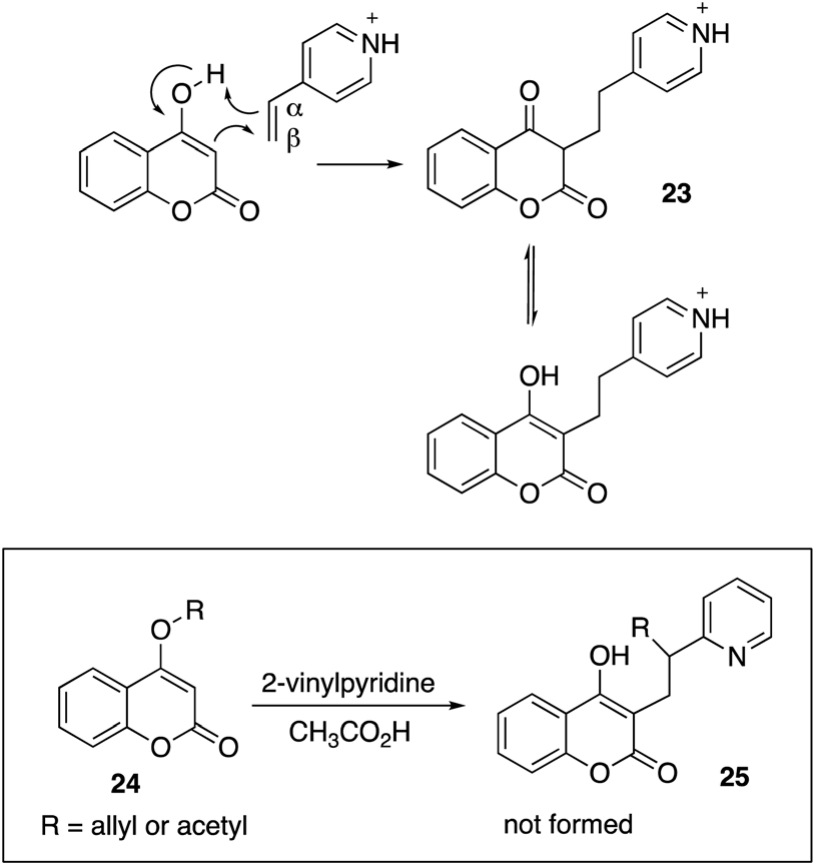
Proposed mechanism for the addition reaction and an unsuccessful application of the chemistry.

As acid-promoted addition reactions, it is suggested that the acid protonates the N-heterocycle and enhances the electrophilic reactivity of the vinyl group (Scheme 2). We propose a mechanism involving nucleophilic attack of the enol group at the electrophilic vinyl group. As the enol transfers electron density into the vinyl group, negative charge accumulates at the α-carbon. This leads to a simultaneous proton transfer to the α-carbon – giving intermediate 23 which rapidly isomerizes to the pyridinium salt of the observed product. Working from the proposed mechanism, we sought to determine if other electrophiles or groups could be transferred to the α-carbon, besides a simple proton. Compounds 24 were prepared, but unfortunately neither the acetyl or allyl groups were observed to migrate and give products 25.

## Conclusions

In summary, we have found that 4-hydroxycuomarins react with vinyl-substituted N-heterocycles using an acid promoter. The enol groups of 4-hydroxycuomarins are sufficiently nucleophilic to undergo Michael additions to vinyl-substituted pyridines, pyrazine, and 1,2,4-oxadiazole. Similar reactivity has been demonstrated with 4-hydroxyquinolin-2(1*H*)-one and 4-hydroxy-2*H*-thiochromen-2-one. This work and other recent studies further demonstrates the utility of Michael addition as a useful route to functionalized heterocycles.^7^

## Author contributions

The experimental work was carried out by B. G. and the conceptual work was done by D. A. K.

## Conflicts of interest

There are no conflicts to declare.

## Supplementary Material

RA-013-D3RA00251A-s001
