# Supplementary material for: Synthesis of warfarin analogs: conjugate addition reactions of alkenyl-substituted N-heterocycles with 4-hydroxycoumarin and related substrates
Source: RSC Adv. 2023 Feb 6;13(7):4754–6. doi: 10.1039/d3ra00251a (PMC9900474; doi:10.1039/d3ra00251a)

Electronic Supplementary Material (ESI) for RSC Advances.
This journal is © The Royal Society of Chemistry 2023

Synthesis of warfarin analogs: conjugate addition reactions of alkenyl-substituted *N*-heterocycles with 4-hydroxycoumarin and related substrates.

Benjamin Goka and Douglas A. Klumpp

Department of Chemistry and Biochemistry

Northern Illinois University

DeKalb, IL 60115

dklumpp@niu.edu

Pages	Content
1	Index
2-6	Experimental procedures and characterization data
7-44	NMR spectra of new compounds

General Methods

Chemical reagents and solvents were purchased from commercial suppliers and used as received. Reactions were performed in dry glassware using an inert atmosphere. Flash chromatography was done using 60 Å silica gel. Products were characterized by ^{1}H and ^{13}C NMR spectroscopy using Bruker Avance III 300 or 500 MHz NMR spectrometers. Chemical shifts were referenced to NMR solvent signals. High-resolution mass spectra were obtained from a commercial analytical laboratory utilizing a mass spectrometer equipped with a time-of-flight (TOF) mass analyzer.

General Procedure. To a solution of 4-hydroxy-2*H*-chromen-2-one (0.35 g, 2.15 mmol) and vinyl-substituted *N*-heterocycle (3.66 mmol) in acetonitrile (30 mL), glacial acetic acid (0.12 mL, 2.15 mmol) is added dropwise. The resulting solution is stirred at 70 °C until TLC analysis no longer shows the presence of the starting material, 4-hydroxy-2*H*-chromen-2-one. After cooling, the mixture is allowed to sit as the product crystallizes from solution. The resulting solid crystals are filtered off, rinsed once with cold acetonitrile, and dried under a vacuum.

4-Hydroxy-3-(2-(pyridin-2-yl)ethyl)-2H-chromen-2-one (3). Using the general procedure, 4-hydroxy-2*H*-chromen-2-one (0.35 g, 2.15 mmol) provided 4-hydroxy-3-(2-(pyridin-2-yl)ethyl)-2H-chromen-2-one (**3**, 0.397 g, 1.48 mmol, 69%) as a white solid. R_f = 0.19 (1:1, EtOAc:hexanes). MP 151-153 °C. ^{1}H NMR (500 MHz, d_6-DMSO) δ 8.57 (dd, J = 5.0, 1.0 Hz, 1H), 7.91 (dd, J = 7.5, 1.0 Hz, 1H), 7.86-7.82 (m, 1H), 7.60-7.56 (m, 1H), 7.42 (d, J = 8.0 Hz, 1H), 7.36-7.32 (m, 3H), 3.10 (t, J = 7.0 Hz, 2H), 2.91 (t, J = 65 Hz, 2H). $^{13}C\{^{1}H\}$ NMR (300 MHz, d_6-DMSO) δ 163.4, 162.0, 160.4, 152.5, 147.7, 138.3, 131.9, 124.4, 124.1, 123.7, 122.4, 117.4, 116.4, 104.8, 35.8, 22.5. HRMS m/z $[M + H]^+$ calcd for $C_{16}H_{14}NO_3$ 268.0974; found 268.0980.

6-Bromo-4-hydroxy-3-(2-(pyridin-2-yl)ethyl)-2H-chromen-2-one (4). Using the general procedure, 6-bromo-4-hydroxy-2H-chromen-2-one (0.35 g, 1.45 mmol) and 2-vinylpyridine (0.27 mL, 2.46 mmol) provided 6-bromo-4-hydroxy-3-(2-(pyridin-2-yl)ethyl)-2H-chromen-2-one (**4**, 0.348 g, 1.00 mmol, 69%) as white solid. R_f = 0.11 (1:1, EtOAc:hexanes). MP 215-218 °C. ^{1}H NMR (300 MHz, d_6-DMSO) δ 8.60-8.58 (m, 1H), 8.00 (s, 1H), 7.99-7.86 (m, 1H), 7.73-7.70 (m, 1H), 7.46 (d, J = 8.1 Hz, 1H), 7.42-7.38 (m, 1H), 7.32 (d, J = 9.0 Hz, 1H), 3.11 (t, J = 6.6 Hz, 2H), 2.89 (t, J = 6.9 Hz, 2H). $^{13}C\{^{1}H\}$ NMR (300 MHz, d_6-DMSO) δ 163.0, 161.7, 160.0, 151.6, 147.1, 139.0, 134.3, 125.9, 124.7, 122.6, 119.8, 118.9, 115.9, 105.1, 35.7, 22.5. HRMS m/z $[M + H]^+$ calcd for $C_{16}H_{13}BrNO_3$ 346.0073; found 346.0076.

6-Chloro-4-hydroxy-3-(2-(pyridin-2-yl)ethyl)-2H-chromen-2-one (5). Using the general procedure, 6-chloro-4-hydroxy-2*H*-chromen-2-one (0.35 g, 1.78 mmol) and 2-vinylpyridine (0.33 mL, 3.03 mmol) provided 6-chloro-4-hydroxy-3-(2-(pyridin-2-yl)ethyl)-2H-chromen-2-one (**5**, 0.459 g, 1.53 mmol, 86%) as white solid. R_f = 0.14 (1:1, EtOAc:hexanes). MP 217-220 °C. ^{1}H NMR (300 MHz, d_6-DMSO) δ 8.59-8.57 (m, 1H), 7.91-7.85 (m, 2H), 7.61-7.57 (m, 1H), 7.47-7.36 (m, 3H), 3.11 (t, J = 6.6 Hz, 2H), 2.89 (t, J = 6.6 Hz, 2H). $^{13}C\{^{1}H\}$ NMR (300 MHz, d_6-DMSO) δ 163.1, 161.7, 160.0, 151.2, 147.1, 139.0, 131.5, 128.1, 124.7, 122.9, 122.6, 119.3, 118.6, 105.2, 35.7, 22.5. HRMS m/z $[M + H]^+$ calcd for $C_{16}H_{13}ClNO_3$ 302.0578; found 302.0579.

4-Hydroxy-6-methyl-3-(2-(pyridin-2-yl)ethyl)-2H-chromen-2-one (6). Using the general procedure, 4-hydroxy-6-methyl-2H-chromen-2-one (0.162 g, 0.9 mmol) and 2-vinylpyridine (0.17 mL, 1.6 mmol) provided 4-hydroxy-6-methyl-3-(2-(pyridin-2-yl)ethyl)-2H-chromen-2-one (**6**, 0.179 g, 0.56 mmol, 62%) as a white solid. R_f = 0.23 (1:1, EtOAc:hexanes). MP 168-170°C. ^{1}H NMR (500 MHz, d_6-DMSO) δ 8.56 (dd, J = 5.0, 1.0 Hz, 1H), 7.85-7.81 (m, 1H), 7.69 (d, J = 1 Hz, 1H), 7.42-7.33 (m, 3H), 7.23 (d, J = 8. Hz, 1H) 3.09 (t, J = 6.5 Hz, 2H), 2.90 (t, J = 6.5 Hz, 2H), 2.37 (s, 3H). ^{13}C{^{1}H} NMR (300 MHz, d_6-DMSO) δ 163.5, 161.9, 160.4, 150.7, 147.7, 138.3, 133.3, 132.7, 124.4, 123.3, 122.4, 117.1, 116.2, 104.8, 35.9, 22.5, 20.9. HRMS m/z [M + H]$^+$ calcd for $C_{17}H_{16}NO_3$ 282.1130; found 282.1131.

6,8-Dichloro-4-hydroxy-3-(2-(pyridin-2-yl)ethyl)-2H-chromen-2-one (7). Using the general procedure, 6,8-dichloro-4-hydroxy-2H-chromen-2-one (0.15 g, 0.65 mmol) and 2-vinylpyridine (0.08 mL, 0.76 mmol) provided 6,8-dichloro-4-hydroxy-3-(2-(pyridin-2-yl)ethyl)-2H-chromen-2-one (**7**, 0.18 g, 0.533 mmol, 82%) as orange solid. R_f = 0.69 (1:1 MeOH:EtOAc). MP >250°C. ^{1}H NMR (500 MHz, d_6-DMSO) δ 8.63 (d, J = 5 Hz, 1H), 7.98 (t, J = 7.5 Hz, 1 H), 7.83 (d, J = 14 Hz, 2H), 7.55 (d, J = 7.5 Hz, 1H), 7.48 (t, J = 5.5 Hz, 1H), 3.16 (t, J = 5.5 Hz, 2H), 2.90 (t, J = 5.0 Hz, 2H). ^{13}C {^{1}H} NMR (500 MHz, d_6-DMSO) δ 162.9, 162.3, 159.6, 147.3, 146.2, 140.1, 130.9, 127.8, 125.2, 123.0, 122.3, 121.3, 121.2, 104.3, 35.4, 22.5. HRMS m/z [M + H]$^+$ calcd for $C_{16}H_{13}NO_3$ 302.0578; found 302.0579. HRMS m/z [M + H]$^+$ calcd for $C_{16}H_{12}Cl_2NO_3$ 336.0189; found 336.0186.

4-Hydroxy-7-methoxy-3-(2-(pyridin-2-yl)ethyl)-2H-chromen-2-one (8). Using the general procedure, 4-hydroxy-6-methoxy-2H-chromen-2-one (0.20 g, 1.0 mmol) and 2-vinylpyridine (0.18 mL, 1.7 mmol) provided 4-hydroxy-6-methoxy-3-(2-(pyridin-2-yl)ethyl)-2H-chromen-2-one (**8**, 0.277 g, 0.89 mmol) as a yellow solid. R_f = 0.14 (1:1 EtOAc:hexanes). MP 166-168°C. ^{1}H NMR (500 MHz, d_6-DMSO) δ 8.55 (d, J = 4.0 Hz, 1H), 7.82-7.79 (m, 2H), 7.39 (d, J = 7.5 Hz, 1H), 7.32 (t, J = 6.0 Hz, 1H), 6.93-6.91 (m, 2H), 3.84 (s, 3H), 3.06 (t, J = 7.0 Hz, 2 H), 2.88 (t, J = 7.0 Hz, 2 H); ^{13}C{H} NMR (500 MHz, d_6-DMSO) δ 163.7, 162.5, 162.0, 160.6, 154.2, 147.9, 138.1, 124.8, 124.2, 122.3, 112.1, 110.3, 102.3, 100.7, 56.2, 36.0, 22.5. HRMS m/z [M + H]$^+$ calcd for $C_{17}H_{16}NO_4$ 298.1074; found 298.1074.

4-Hydroxy-3-(2-(5-nitropyridin-2-yl)ethyl)-2H-chromen-2-one (9). Using the general procedure, 5-hydroxy-2H-chromen-2-one (0.30 g, 1.9 mmol) and 5-nitro-2-vinylpyridine (0.278 g, 1.9 mmol) provided 4-hydroxy-3-(2-(5-nitropyridin-2-yl)ethyl)-2H-chromen-2-one (**9**, 0.27 g, 0.893 mmol, 47%) as white solid. R_f = 0.22 (1:1 EtOAc:hexanes). MP 198-201°C. ^{1}H NMR (500 MHz, d_6-DMSO) δ 9.29 (d, J = 7 Hz, 1H), 8.52 (dd, J = 8.5, 2.5 Hz, 1H), 7.93 (dd, J = 8.0, 1.5 Hz, 1H), 7.62-7.58 (m, 2H), 7.37-7.35 (m, 2H), 3.10-3.07 (m, 2H), 2.97-2.94 (m, 2H). ^{13}C{H} NMR (500 MHz, d_6-DMSO) δ 168.2, 163.1, 160.8, 152.3, 144.4, 143.1, 132.2, 132.1, 124.3, 124.0, 123.7, 116.7, 116.6, 104.2, 36.3, 23.8. HRMS m/z [M + H]$^+$ calcd for $C_{16}H_{13}N_2O_5$ 313.0823; found 313.0819.

4-Hydroxy-3-(2-(pyridin-4-yl)ethyl)-2H-chromen-2-one (10). Using the general procedure, 4-hydroxy-2H-chromen-2-one (0.35 g, 2.15 mmol) and 4-vinylpyridine (0.4 mL, 3.67 mmol) provided 4-hydroxy-3-(2-(pyridin-4-yl)ethyl)-2H-chromen-2-one (**10**, 0.438 g, 1.63 mmol, 76%) as a white solid. R_f = 0.15 (1:4, MeOH:EtOAc). MP 235-240°C. ^{1}H NMR (500 MHz, d_6-DMSO) δ 8.45 (d, J = 6Hz, 2H), 7.93 (dd, J = 7.5, 1 Hz, 1H), 7.59 (t, J = 8.5 Hz, 1H), 7.36-7.32 (m, 2H), 7.28 (d, J =

6 Hz, 2H), 2.85-2.82 (m, 2H), 2.79-2.76 (m, 2H). $^{13}C\{^1H\}$ NMR (500 MHz, d_6-DMSO) δ 163.2, 161.1, 152.4, 151.0, 149.7, 132.0, 124.4, 124.2, 123.7, 117.0, 116.5, 103.8, 33.2, 24.9. HRMS m/z [M + H]$^+$ calcd for $C_{16}H_{14}NO_3$ 268.0968; found 268.0971.

6-Bromo-4-hydroxy-3-(2-(pyridin-4-yl)ethyl)-2H-chromen-2-one (11). Using the general procedure, 6-bromo-4-hydroxy-2H-chromen-2-one (0.21 g, 0.86 mmol) and 4-vinylpyridine (0.16 mL, 1.46 mmol) provided 6-bromo-4-hydroxy-3-(2-(pyridin-4-yl)ethyl)-2H-chromen-2-one, (**11**, 0.212 g, 0.61 mmol, 71%) as yellow solid. R_f = 0.20 (1:4, MeOH:EtOAc). MP >250°C. ^1H NMR (300 MHz, d_6-DMSO) δ 8.47 (d, J = 5.7 Hz, 2H), 8.04 (d, J = 2.4 Hz, 1H), 7.74 (dd, J = 8.7, 2.4 Hz, 1H), 7.34-7.30 (m, 3H), 2.84-2.73 (m, 4H). $^{13}C\{^1H\}$ NMR (300 MHz, d_6-DMSO) δ 162.9, 160.5, 151.8, 151.5, 149.1, 134.4, 126.0, 124.6, 119.3, 118.9, 116.0, 104.3, 33.2, 25.0. HRMS m/z [M + H]$^+$ calcd for $C_{16}H_{13}BrNO_3$ 346.0073, found 346.0075.

6-Chloro-4-hydroxy-3-(2-(pyridin-4-yl)ethyl)-2H-chromen-2-one (12). Using the general procedure, 6-chloro-4-hydroxy-2H-chromen-2-one (0.35 g, 1.78 mmol) and 4-vinylpyridine (0.33 mL, 3.03 mmol) provides 6-chloro-4-hydroxy-3-(2-(pyridin-4-yl)ethyl)-2H-chromen-2-one (**12**, 0.439 g, 1.46 mmol, 82%) as yellow solid. R_f = 0.20 (1:4, MeOH:EtOAc). MP 254-256°C. ^1H NMR (500 MHz, d_6-DMSO) δ 8.47 (d, J = 5.7 Hz, 2H), 7.91 (d, J = 2.4 Hz, 1H), 7.62 (dd, J = 6.3, 2.4 Hz, 1H), 7.39 (d, J = 8.7 Hz, 1H), 7.32-7.30 (m, 2H), 2.83-2.76 (m, 4H). $^{13}C\{^1H\}$ NMR (500 MHz, d_6-DMSO) δ 162.9, 160.8, 151.7, 151.1, 149.2, 131.6, 128.2, 124.5, 123.1, 118.9, 118.6, 104.2, 33.2, 25.0. HRMS m/z [M + H]$^+$ calcd for $C_{16}H_{13}ClNO_3$ 302.0578, found 302.0583.

6,8-Dichloro-4-hydroxy-3-(2-(pyridin-4-yl)ethyl)-2H-chromen-2-one (13). Using the general procedure, 6,8-dichloro-4-hydroxy-2H-chromen-2-one (0.35 g, 1.51 mmol) and 4-vinylpyridine (0.16 mL,1.51 mmol) provided 6,8-dichloro-4-hydroxy-3-(2-(pyridin-4-yl)ethyl)-2H-chromen-2-one (**13**, 0.299 g, 0.89 mmol, 59%) as yellow solid using silica gel chromatography (1:1, ethyl acetate:methanol). R_f = 0.42 (1:1 MeOH:EtOAc). MP >250°C. ^1H NMR (500 MHz, d_6-DMSO) δ 8.41 (dd, J = 4.5, 1.5 Hz, 2H), 7.70 (d, J = 2.5 Hz, 1H), 7.61 (d, J = 2.5 Hz, 1H), 7.24 (d, J = 5.5 Hz, 2H), 2.74-2.71 (m, 2H), 2.65-2.62 (m, 2H). $^{13}C\{^1H\}$ NMR (500 MHz, d_6-DMSO) δ 164.1, 160.1, 151.6, 149.7, 138.1, 129.9, 124.3, 123.5, 121.0, 117.0, 115.1, 109.9, 33.6, 24.6. HRMS m/z [M + H]$^+$ calcd for $C_{16}H_{12}Cl_2NO_3$ 336.0186; found 336.0191.

4-Hydroxy-7-methoxy-3-(2-(pyridin-4-yl)ethyl)-2H-chromen-2-one (14). Using the general procedure, 4-hydroxy-6-methoxy-2H-chromen-2-one (0.2 g, 1.0 mmol) and 4-vinylpyridine (0.18 mL, 1.7 mmol) provides 4-hydroxy-6-methoxy-3-(2-(pyridin-4-yl)ethyl)-2H-chromen-2-one, (**14**, 0.159 g, 0.57 mmol) as orange solid. R_f = 0.23 (1:4, MeOH:EtOAc). MP 162-165°C. ^1H NMR (300 MHz, d_6-DMSO) δ 8.45 (s, 2H), 7.84-7.81 (m, 1H), 7.27 (d, J = 4.5 Hz, 2H), 6.94-6.90 (m, 2H), 3.84 (s, 3H), 2.79-2.74 (m, 4H). ^{13}C $\{^1H\}$ NMR (300 MHz, d_6-DMSO) δ 163.6, 162.5, 161.5, 154.1, 151.0, 149.6, 124.8, 124.3, 112.1, 110.0, 101.2, 100.7, 56.2, 33.3, 24.8. HRMS m/z [M + H]$^+$ calcd for $C_{17}H_{16}NO_4$ 298.1074, found 298.1074.

3-(1,2-Di(pyridin-4-yl)ethyl)-4-hydroxy-2H-chromen-2-one (15). Using the general procedure, 4-hydroxy-2H-chromen-2-one (0.35 g, 2.17 mmol) and (*E*)-1,2-di(pyridin-4-yl)ethene (0.396 g, 2.17 mmol) provided 3-(1,2-di(pyridin-4-yl)ethyl)-4-hydroxy-2H-chromen-2-one (**16**, 0.545 g, 1.58

mmol, 73%) as yellow solid using silica gel chromatography (1:2, MeOH:EtOAc). R_f = 0.28 (1:2, MeOH:EtOAc). MP 65-70°C. ^{1}H NMR (500 MHz, d_4-methanol) δ 8.36 (d, J = 5.0 Hz, 2H), 8.30 (d, J = 5.5 Hz, 2H), 7.91 (dd, J = 8.0, 1.5 Hz, 1H), 7.61 (d, J = 6.0 Hz, 2H), 7.43-7.39 (m, 1H), 7.37 (d, J = 6.0, 2H), 7.19-7.14 (m, 1H), 7.12 (d, J = 0.5 Hz, 1H), 5.00-4.97 (m, 1H), 3.89-3.84 (m, 1H), 3.51-3.47 (m, 1H). ^{13}C {^{1}H} NMR (500 MHz, d_4-methanol) δ 174.8, 166.5, 156.1, 153.5, 152.1, 147.9, 147.7, 130.1, 124.8, 124.3, 123.8, 122.3, 122.2, 115.5, 99.7, 36.9, 35.4. HRMS m/z [M + H]$^+$ calcd for $C_{21}H_{17}N_2O_3$ 345.1234, found 345.1236.

4-Hydroxy-3-(2-(3-phenyl-1,2,4-oxadiazol-5-yl)ethyl)-2H-chromen-2-one (17). Using the general procedure, 4-hydroxy-2H-chromen-2-one (0.35 g, 2.16 mmol) and 3-phenyl-5-vinyl-1,2,4-oxadiazole (0.6 mL) provided 4-hydroxy-3-(2-(3-phenyl-1,2,4-oxadiazol-5-yl)ethyl)-2H-chromen-2-one, (**18**, 0.211 g, 0.62 mmol, 29%) as a white solid. R_f = 0.32 (3:7, MeOH:EtOAc). MP 208-211°C. ^{1}H NMR (500 MHz, d_6-DMSO) δ 8.00-7.94 (m, 3H), 7.62-7.55 (m, 4H), 7.39-7.36 (m, 2H), 3.18 (t, J = 7.5 Hz, 2H), 3.05 (t, J = 8.0 Hz, 2H). ^{13}C {^{1}H} NMR (500 MHz, d_6-DMSO) δ 180.0, 167.9, 163.2, 161.5, 152.4, 132.4, 131.9, 129.7, 127.4, 126.8, 124.4, 123.8, 116.7, 116.6, 102.8, 25.0, 21.7. HRMS m/z [M + H]$^+$ calcd for $C_{19}H_{15}N_2O_4$ 335.1032, found 335.1030.

4-Hydroxy-3-(2-(pyrazin-2-yl)ethyl)-2H-chromen-2-one (18). Using the general procedure, 4-hydroxy-2H-chromen-2-one (0.2 g, 1.23 mmol) and 2-vinylpyrazine (0.21 mL, 2.09 mmol) provided 4-hydroxy-3-(2-(pyrazin-2-yl)ethyl)-2H-chromen-2-one (**19**, 0.131 g, 0.47 mmol, 39%) as a brown solid using silica gel chromatography (3:7, MeOH:EtOAc). R_f = 0.62 (3:7, MeOH:EtOAc). MP 154-160°C. ^{1}H NMR (500 MHz, d_6-DMSO) δ 8.57-8.47 (m, 3H), 7.92 (dd, J = 8.0, 1.0 Hz, 1H), 7.62-7.59 (m, 1H), 7.37-7.34 (m, 2H), 3.00-2.92 (m, 4H). ^{13}C{^{1}H} NMR (500 MHz, d_6-DMSO) δ 163.1, 160.7, 156.8, 152.3, 145.1, 144.2, 142.8, 132.2, 124.3, 123.6, 116.68, 116.61, 104.3, 33.4, 23.7. HRMS m/z [M + H]$^+$ calcd for $C_{15}H_{13}N_2O_3$ 269.0921, found 269.0918.

4-Hydroxy-3-(2-(pyridin-2-yl)ethyl)quinolin-2(1H)-one (19). Using the general procedure, 4-hydroxyquinolin-2(1H)-one (0.35 g, 2.17 mmol) and 2-vinylpyridine (0.37 mL, 3.69 mmol) provided 4-hydroxy-3-(2-(pyridin-2-yl)ethyl)quinolin-2(1H)-one, (**20**, 0.267 g, 1.04 mmol, 48%) as a yellow solid. R_f = 0.58 (3:7, MeOH:EtOAc). MP > 250°C. ^{1}H NMR (300 MHz, d_6-DMSO) δ 11.30 (s, 1H), 8.55-8.53 (m, 1H), 7.88-7.85 (m, 1H), 7.78-7.75 (m, 1H), 7.44-7.36 (m, 2H), 7.29-7.23 (m, 2H), 7.16-7.13 (m, 1H), 3.07 (t, J = 6.9 Hz, 2H), 2.96 (t, J = 6.3 Hz, 2H). ^{13}C {^{1}H} NMR (300 MHz, d_6-DMSO) δ 164.0, 161.0, 158.5, 148.2, 138.0, 137.7, 130.2, 124.1, 123.1, 122.1, 121.3, 116.2, 115.2, 111.7, 36.2, 21.9. HRMS m/z [M + H]$^+$ calcd for $C_{16}H_{15}N_2O_2$ 267.1128, found 267.1132.

4-Hydroxy-3-(2-(pyridin-4-yl)ethyl)quinolin-2(1H)-one (20). Using the general procedure, 4-hydroxyquinolin-2(1H)-one (0.35 g, 2.17 mmol) and 2-vinylpyridine (0.37 mL, 3.69 mmol) provided 4-hydroxy-3-(2-(pyridin-2-yl)ethyl)quinolin-2(1H)-one, (**21**, 0.289 g, 1.09 mmol, 50%) as a yellow solid. R_f = 0.93 (1:1, MeOH:EtOAc). MP > 250°C. ^{1}H NMR (500 MHz, d_6-DMSO) δ 11.13 (s, 1H), 8.42 (d, J = 4.0 Hz, 2H), 7.94 (d, J = 7.5 Hz, 1H), 7.42-7.38 (m, 1H), 7.26-7.22 (m, 3H), 7.09 (t, J = 7.5 Hz, 1H), 2.89-2.85 (m, 2H), 2.76-2.73 (m, 2H). ^{13}C {^{1}H} NMR (500 MHz, d_6-DMSO) δ 164.1, 160.1, 151.6, 149.7, 138.1, 129.9, 124.3, 123.5, 121.0, 117.0, 115.1, 109.9, 33.6, 24.6. HRMS m/z [M + H]$^+$ calcd for $C_{16}H_{15}N_2O_2$ 267.1128, found 267.1129.

6-Bromo-4-hydroxy-3-(2-(pyridin-2-yl)ethyl)quinolin-2(1H)-one (21). Using the general procedure, 6-bromo-4-hydroxyquinolin-2(1H)-one (0.25 g, 1.0 mmol) and 2-vinylpyridine (0.19 mL, 1.8 mmol) provided 6-bromo-4-hydroxy-3-(2-(pyridin-2-yl)ethyl)quinolin-2(1H)-one (**22**, 0.224 g,) as a white solid. Rf = 0.78 (1:1, MeOH:EtOAc). MP 223-227°C. ^{1}H NMR (300 MHz, d_6-DMSO) δ 11.41 (s, 1H), 8.55-8.53 (m, 1H), 7.98 (d, J = 2.1 Hz, 1H), 7.81-7.75 (m, 1H), 7.58 (dd, J = 8.7, 2.4 Hz, 1H), 7.38 (d, J = 7.8 Hz, 1H), 7.32-7.27 (m, 1H), 7.20 (d, J = 8.7 Hz, 1H), 3.05 (t, J = 6.9 Hz, 2H), 2.96-2.91 (t, J = 6.0 Hz, 2H). $^{13}C\{^{1}H\}$ NMR (300 MHz, d_6-DMSO) δ 163.8, 160.9, 157.8, 148.1, 137.8, 137.0, 132.7, 125.3, 124.1, 122.1, 118.2, 117.4, 113.1, 112.6, 36.1, 22.0. HRMS m/z $[M + H]^+$ calcd for $C_{16}H_{14}BrN_2O_2$ 348.0239, found 348.0235.

4-Hydroxy-3-(2-(pyridin-2-yl)ethyl)-2H-thiochromen-2-one (22). Using the general procedure, 4-hydroxy-2H-thiochromen-2-one (0.2 g, 1.12 mmol) and 2-vinylpyridine (0.21 mL, 1.91 mmol) provided 4-hydroxy-3-(2-(pyridin-2-yl)ethyl)-2H-thiochromen-2-one, (**23**, 0.129 g, 0.46 mmol, 41%) as white solid. Rf = 0.80 (1:1 MeOH:EtOAc). MP 144-150°C. ^{1}H NMR (300 MHz, d_6-DMSO) δ 8.59-8.58 (m, 1 H), 8.27-8.25 (m, 1 H), 7.91-7.88 (m, 1 H), 7.54-7.52 (m, 2 H), 7.48-7.44 (m, 2H), 7.42-7.39 (m, 1H), 3.20-3.18 (m, 2 H), 3.01-2.99 (m, 2 H). $^{13}C\{^{1}H\}$ NMR (300 MHz, d_6-DMSO) δ 183.0, 164.7, 159.8, 146.7, 139.2, 135.0, 130.4, 127.0, 126.7, 125.7, 125.6, 125.0, 122.8, 117.0, 35.7, 21.7. HRMS m/z $[M + H]^+$ calcd for $C_{16}H_{14}NO_2S$ 284.0740, found 284.0744.

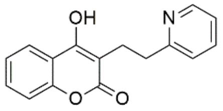

3

^{1}H NMR (300 MHz, d_6-DMSO)

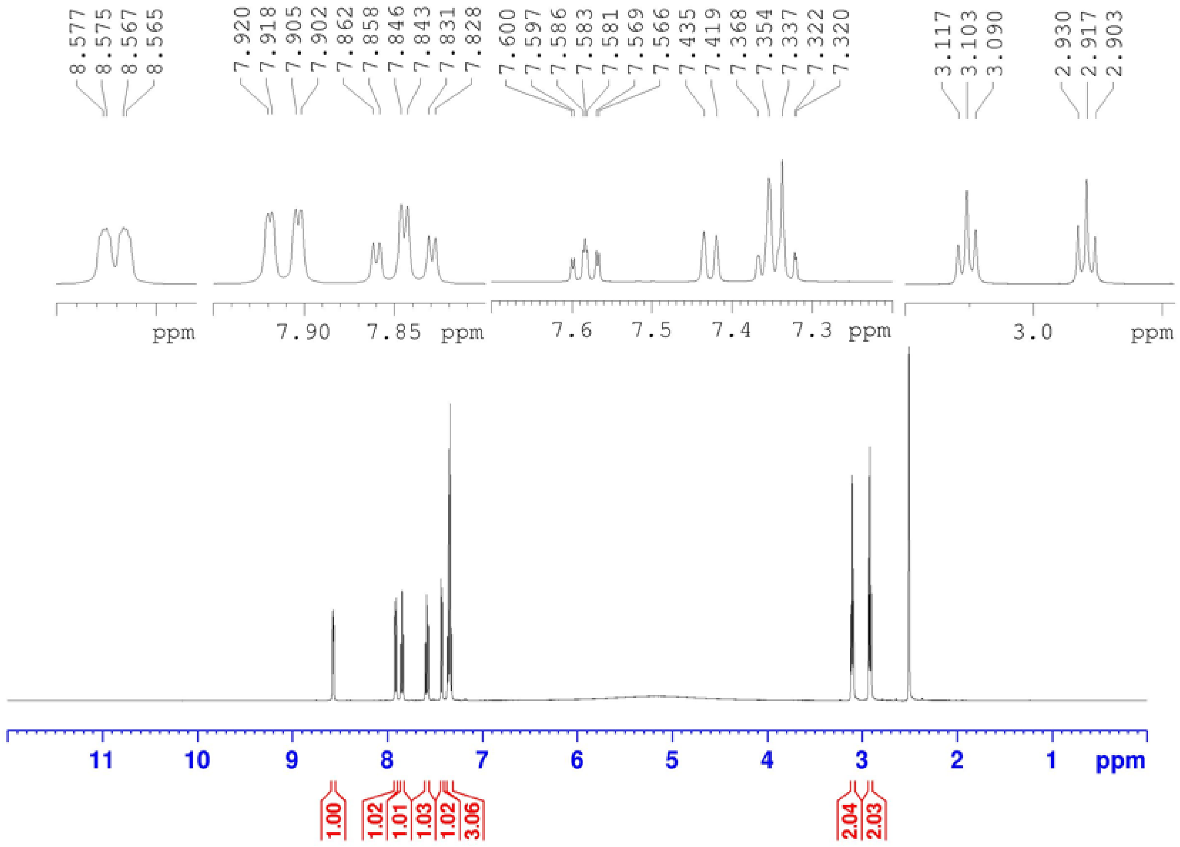

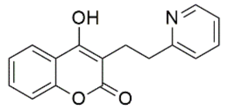

3

^{13}C NMR (300 MHz, d_6-DMSO)

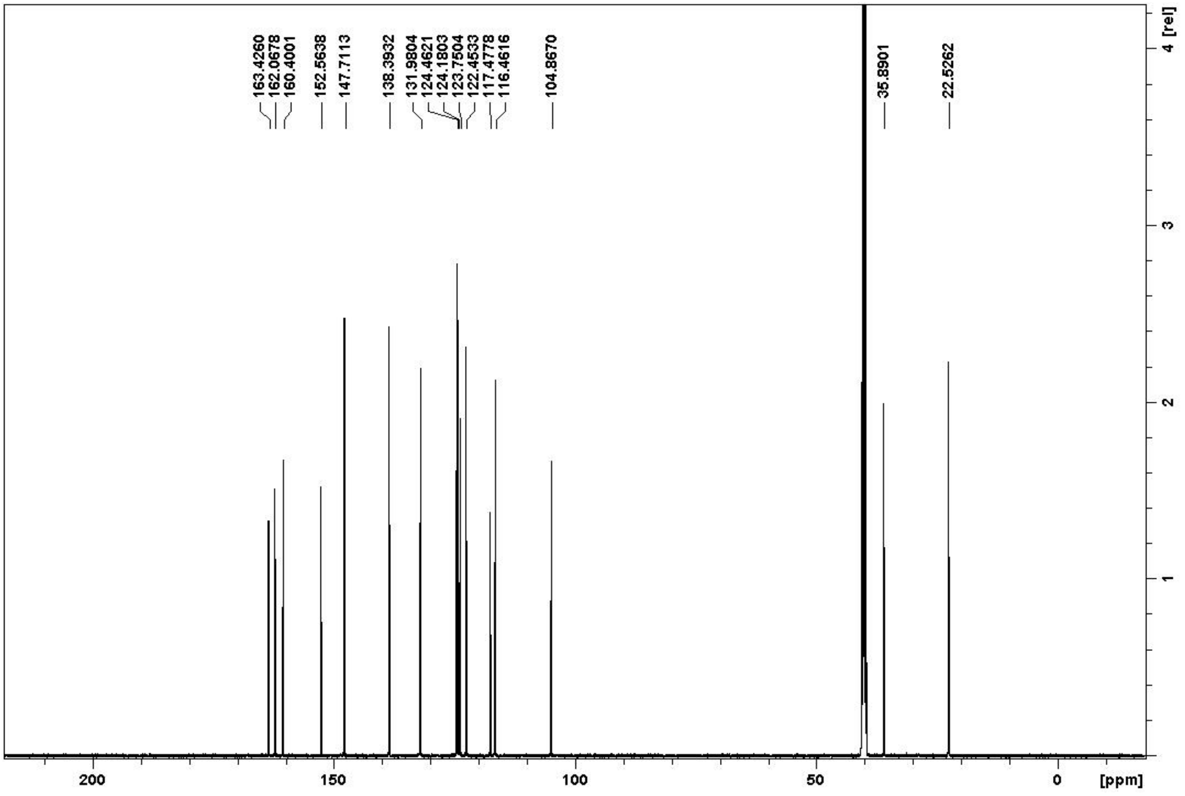

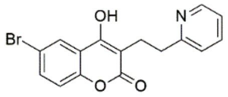

4

^{1}H NMR (300 MHz, d_6-DMSO)

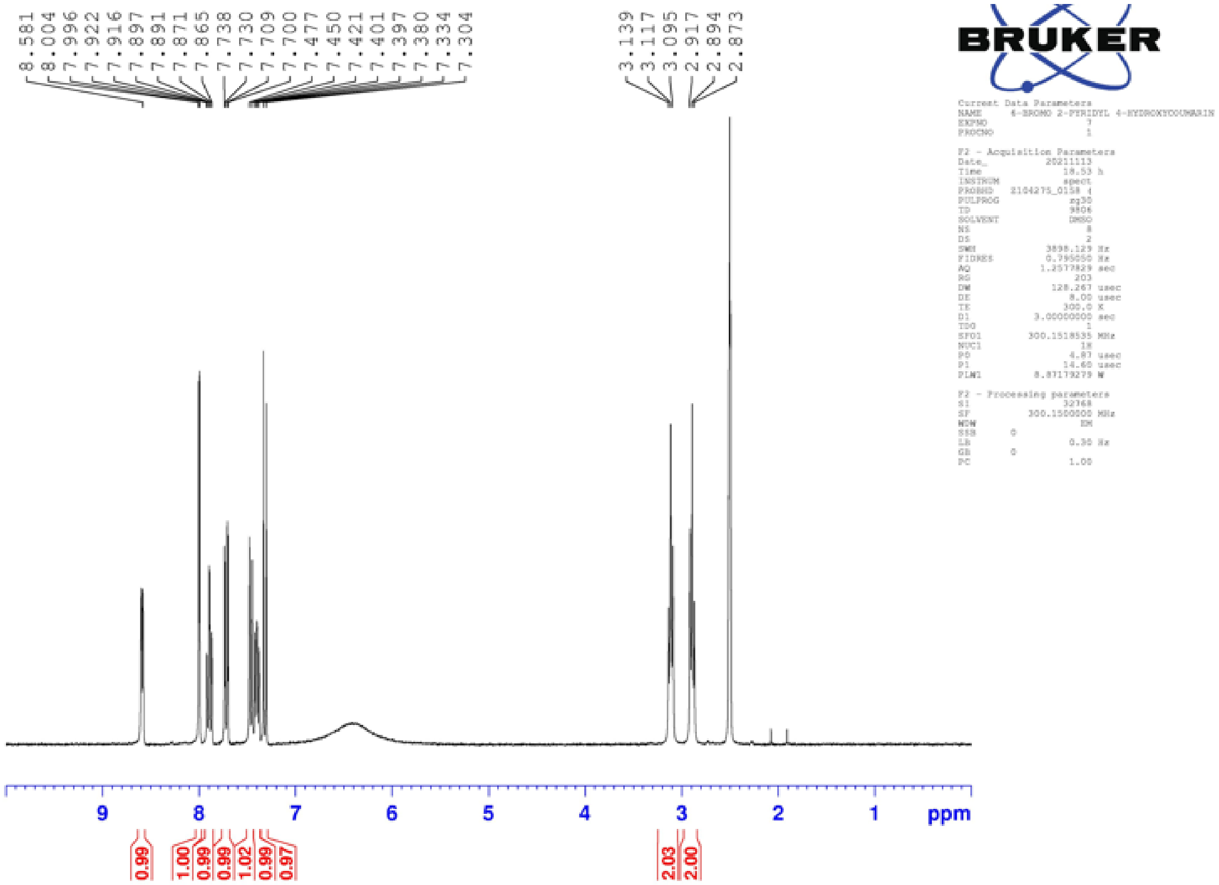

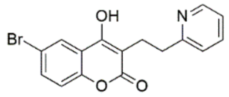

4

^{13}C NMR (300 MHz, d_6-DMSO)

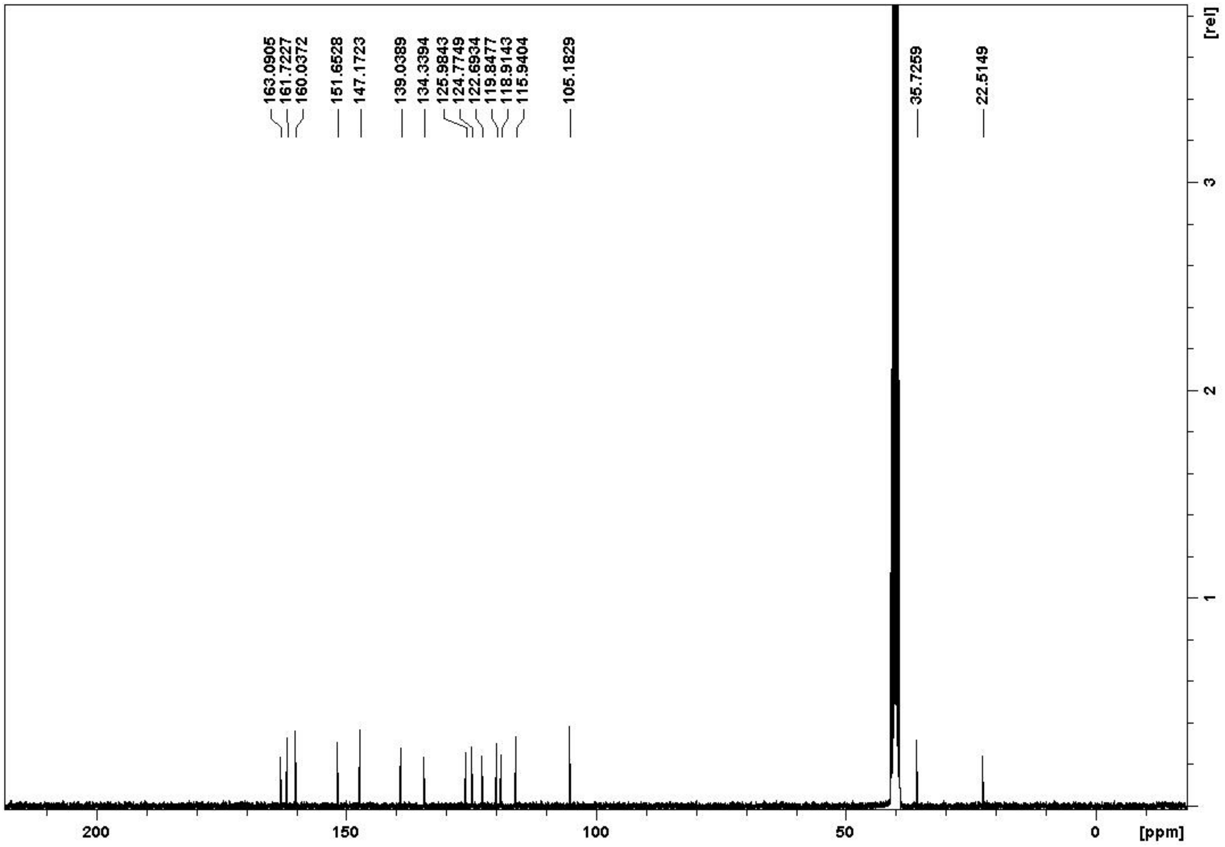

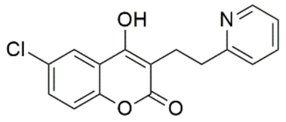

5

^{1}H NMR (300 MHz, d_6-DMSO)

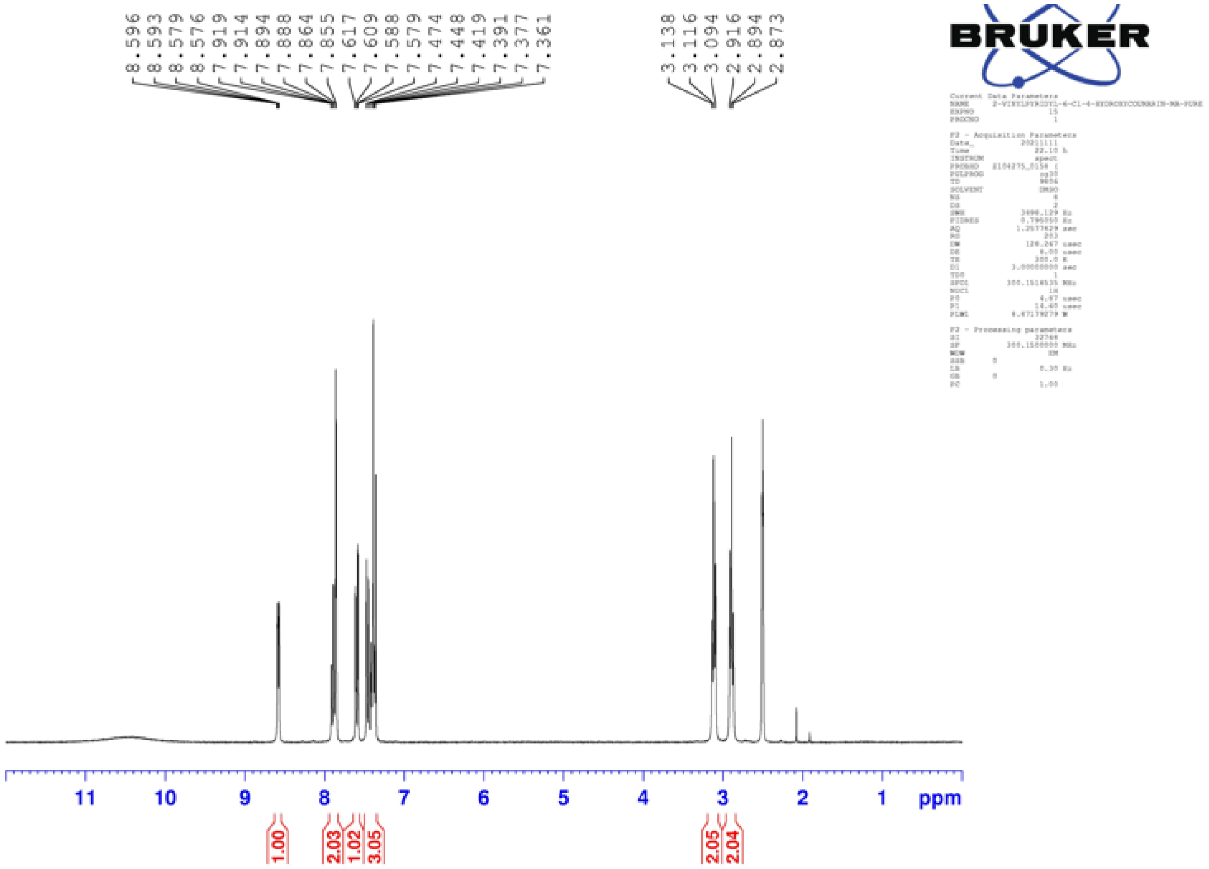

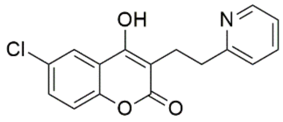

5

^{13}C NMR (300 MHz, d_6-DMSO)

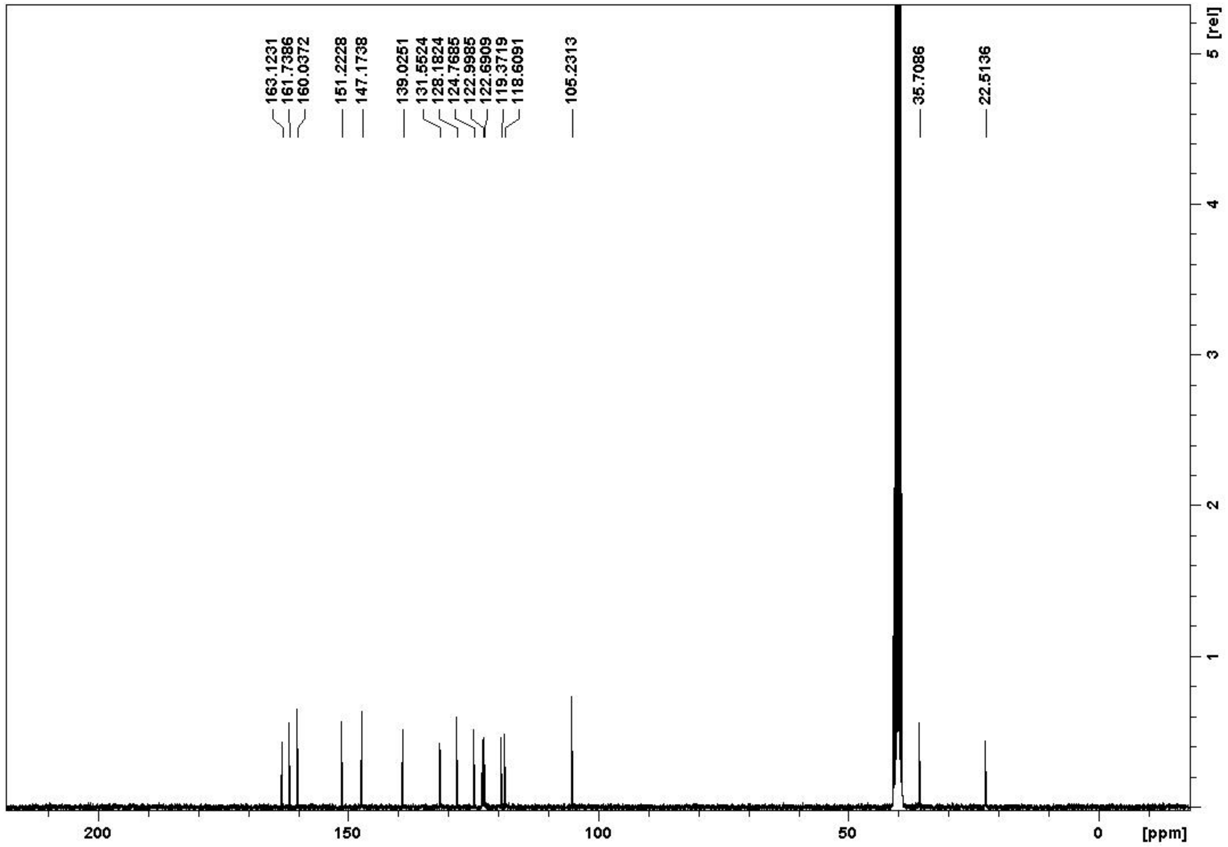

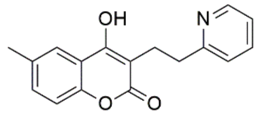

6

^{1}H NMR (300 MHz, d_6-DMSO)

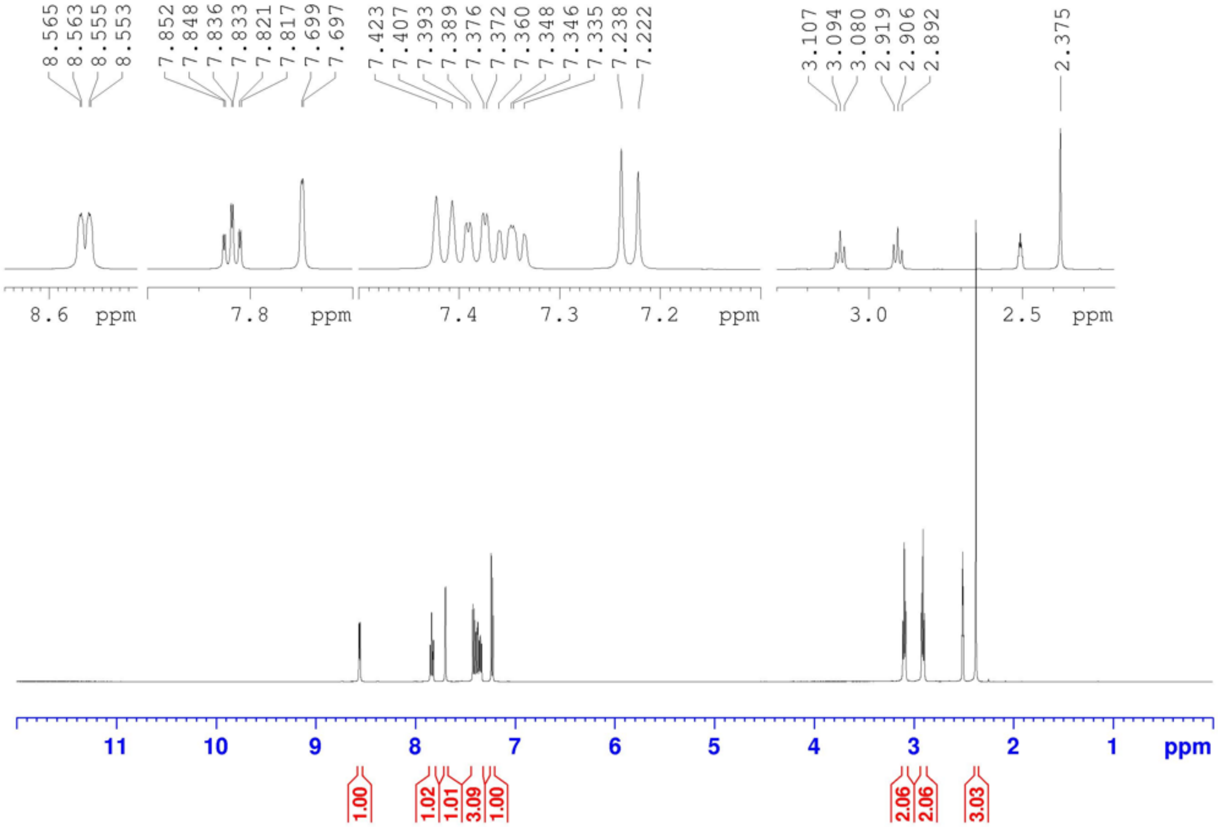

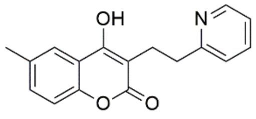

6

^{13}C NMR (300 MHz, d_6-DMSO)

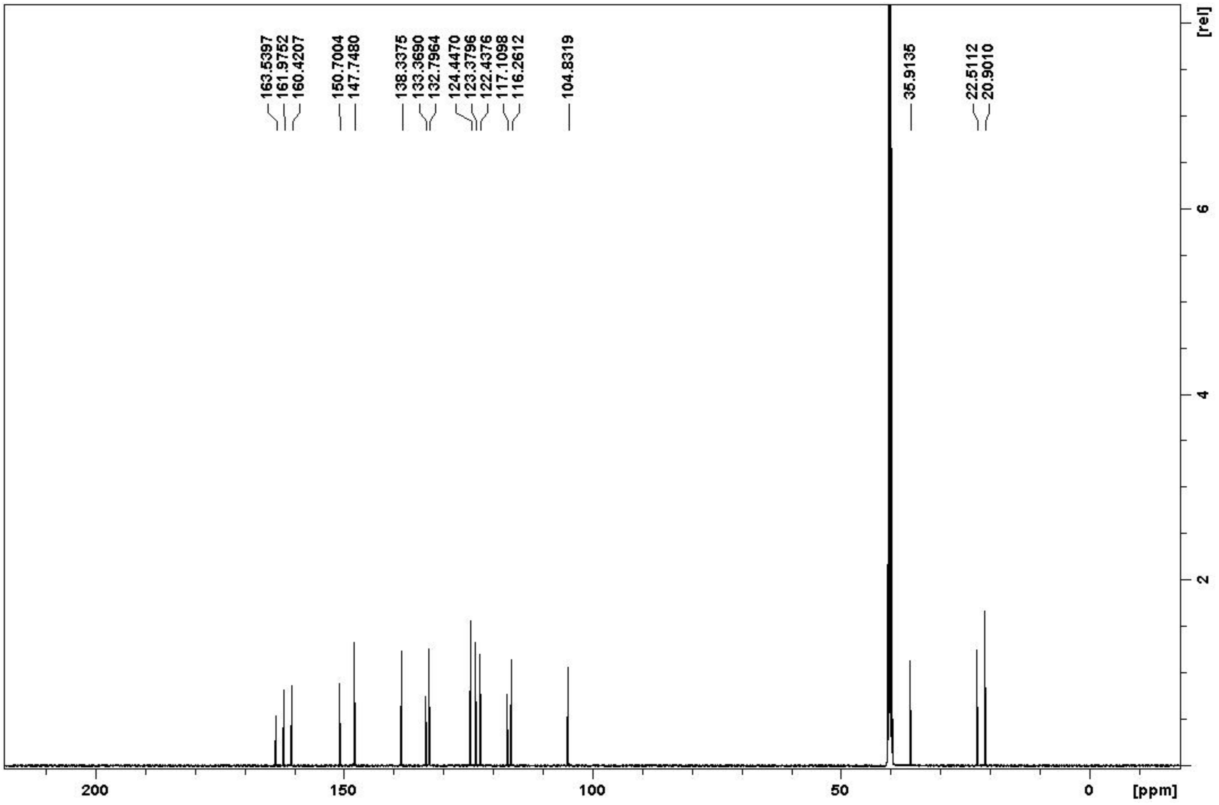

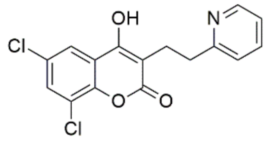

7

^{1}H NMR (500 MHz, d_6-DMSO)

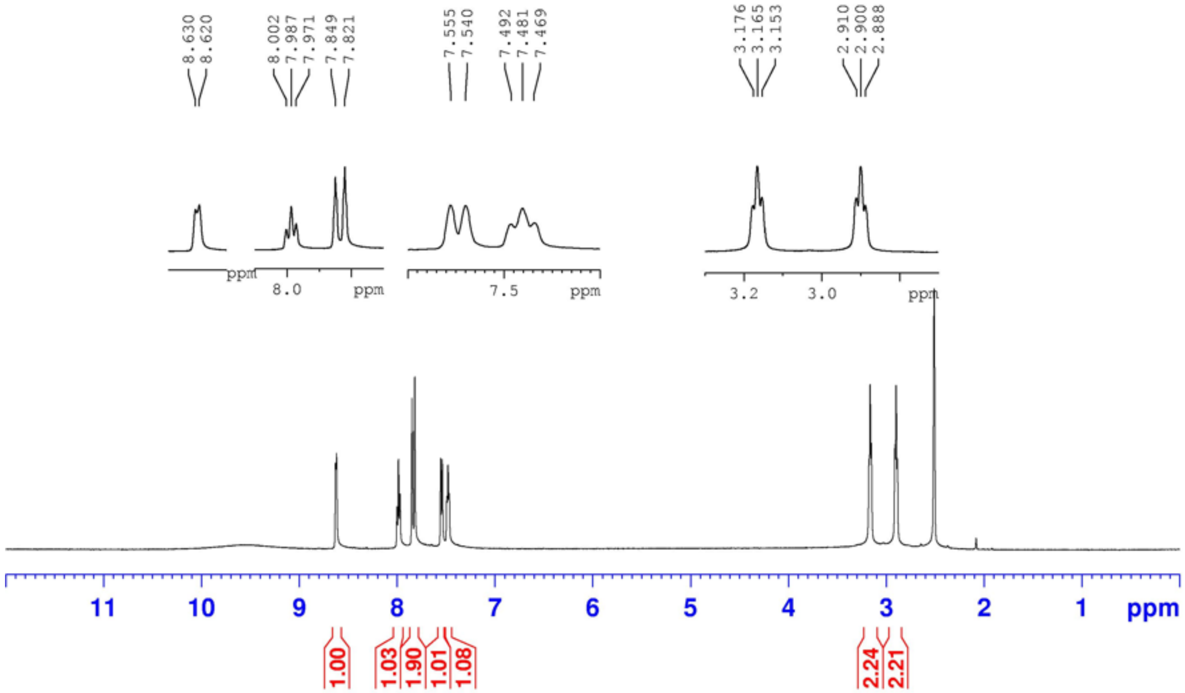

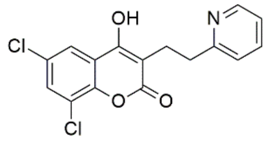

7

^{13}C NMR (500 MHz, d_6-DMSO)

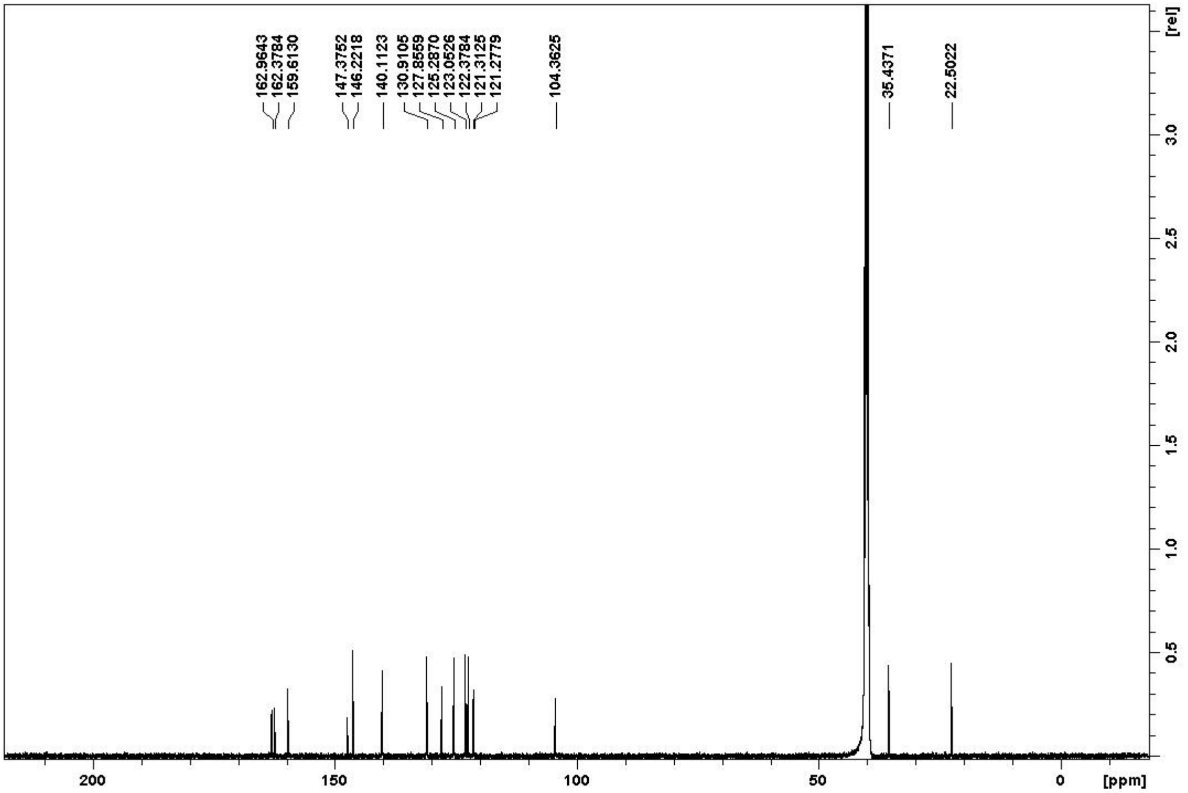

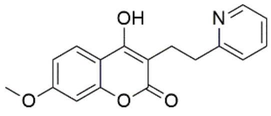

8

^{1}H NMR (500 MHz, d_6-DMSO)

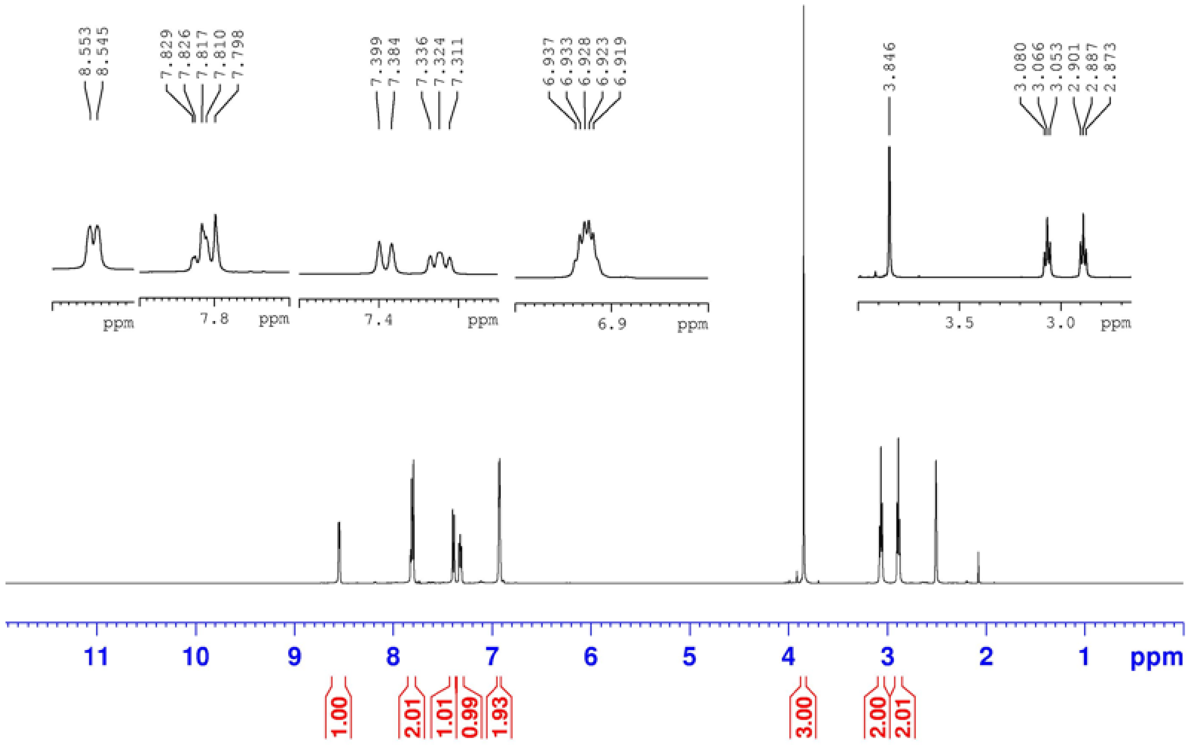

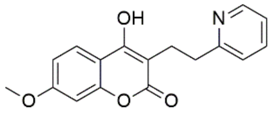

8

^{13}C NMR (500 MHz, d_6-DMSO)

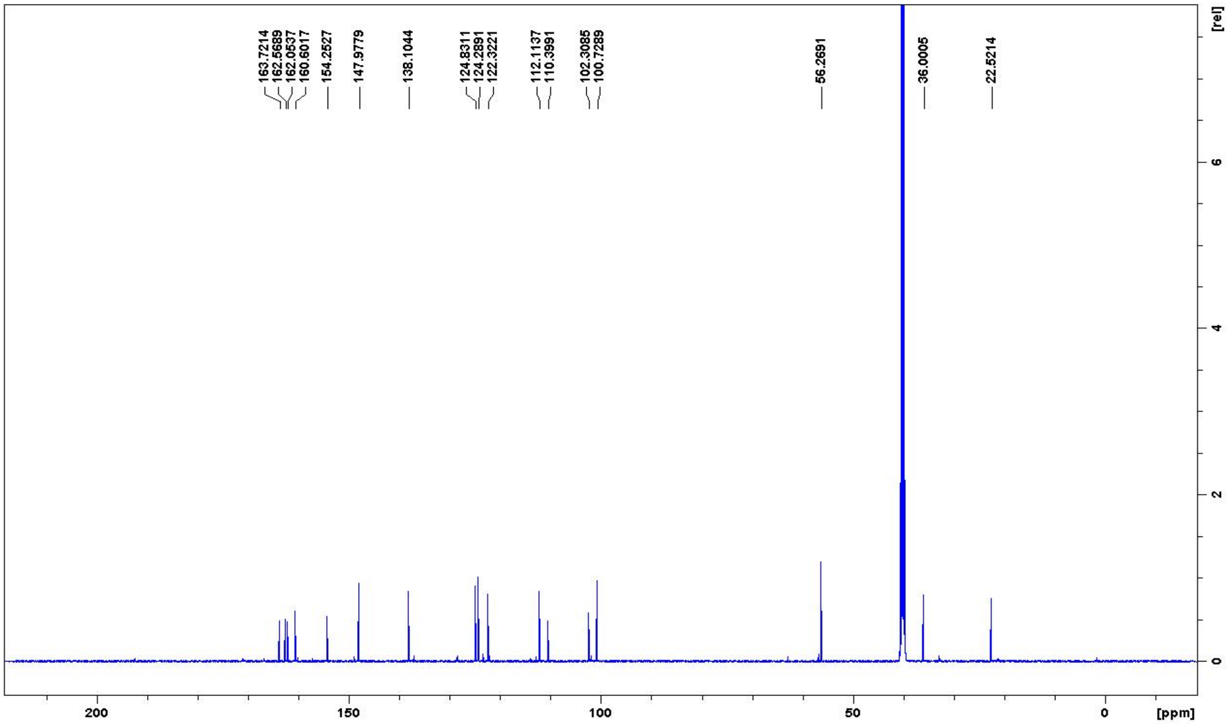

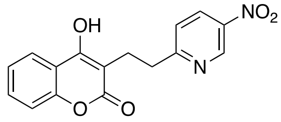

9

^{1}H NMR (500 MHz, d_6-DMSO)

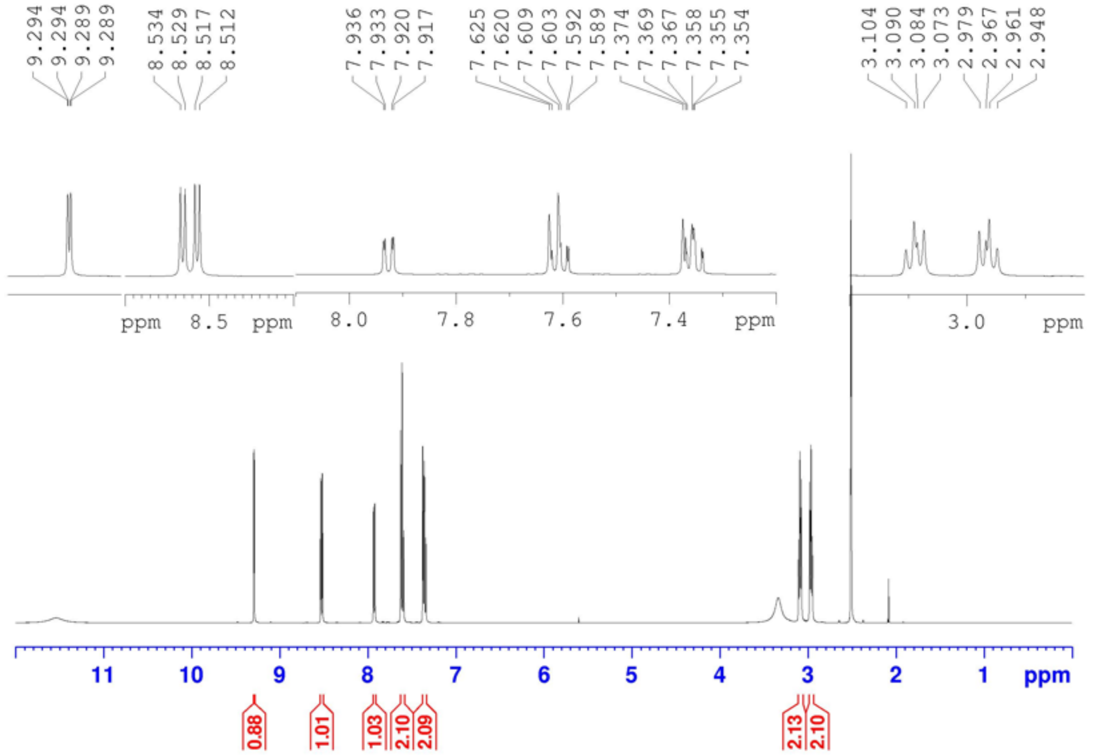

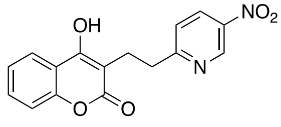

9

^{13}C NMR (500 MHz, d_6-DMSO)

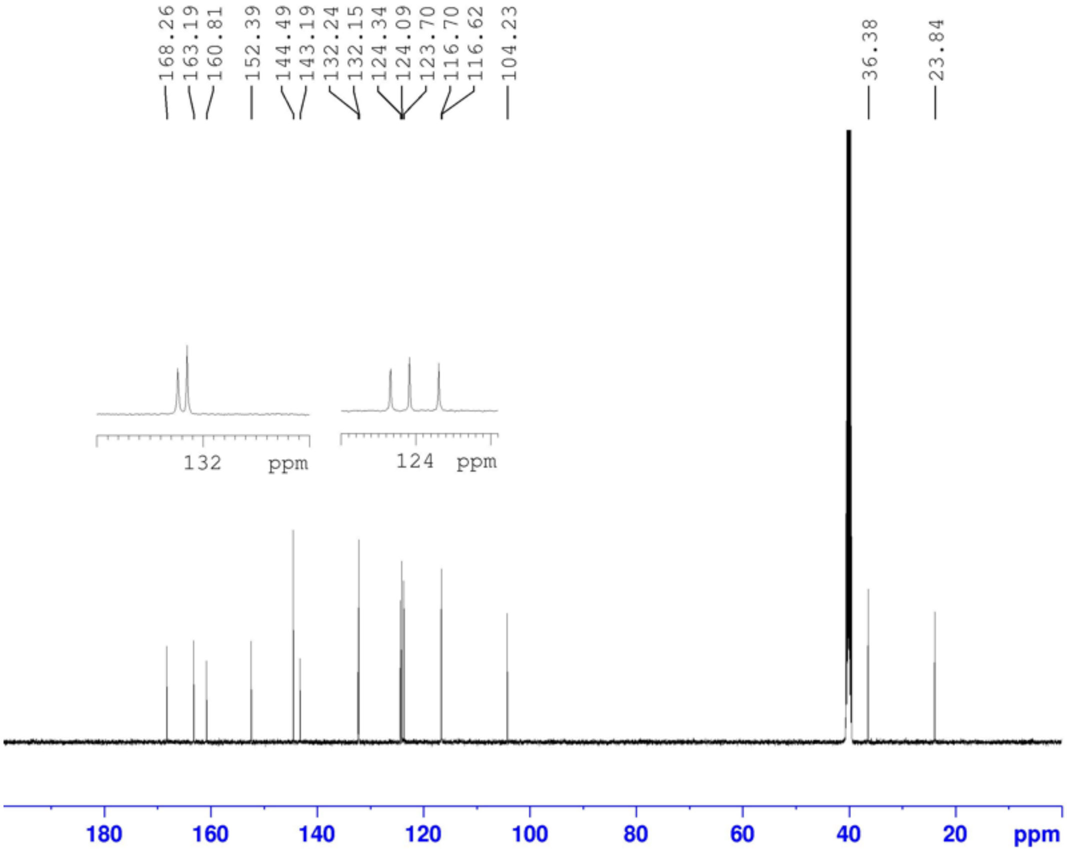

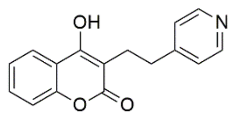

10

^{1}H NMR (500 MHz, DMSO-d_6)

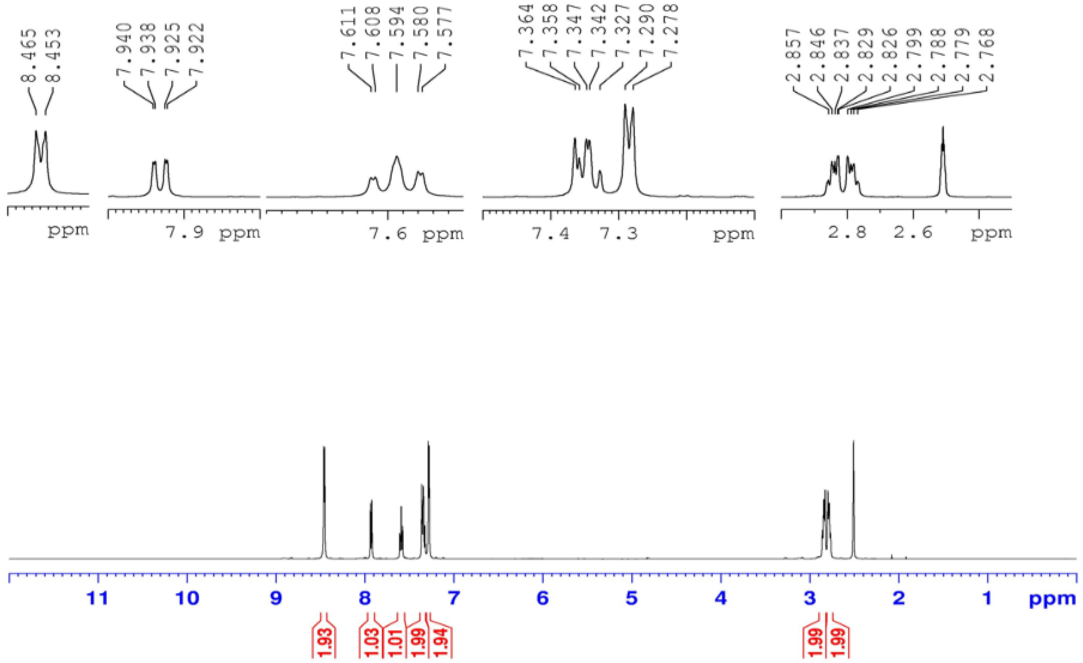

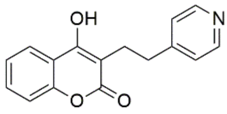

10

^{13}C NMR (500 MHz, DMSO-d$_6$)

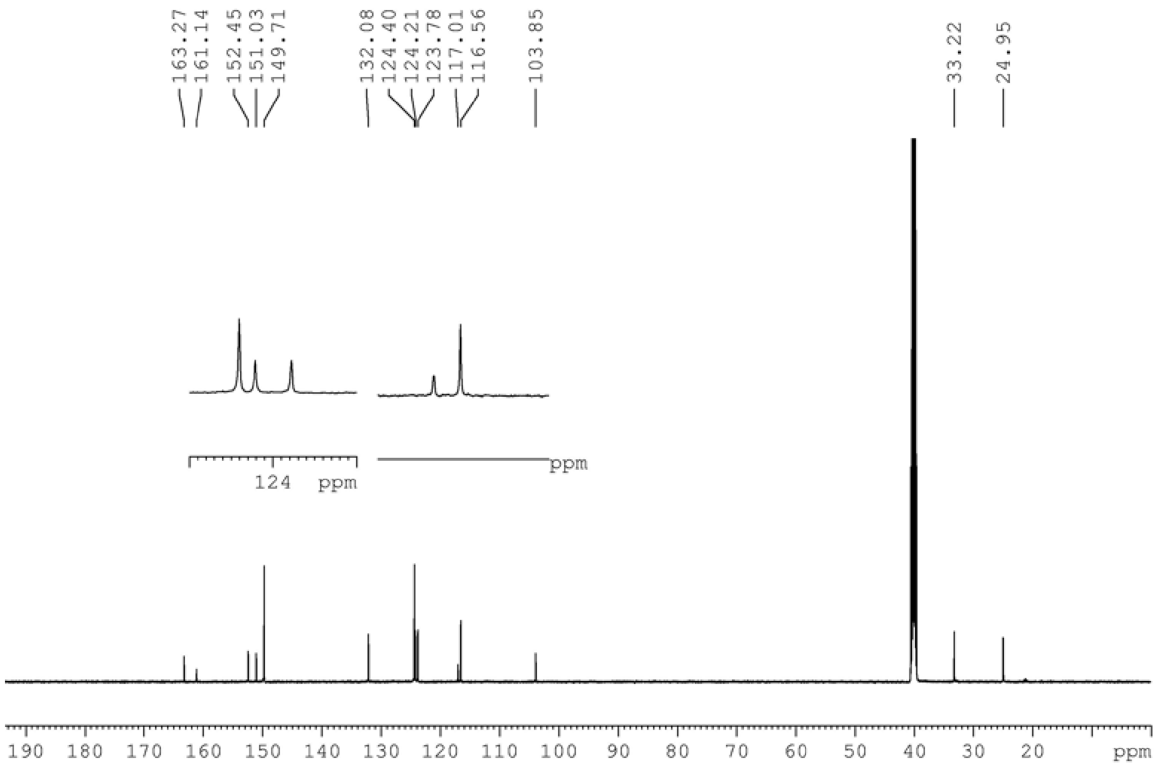

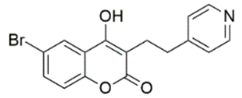

11

^{1}H NMR (300 MHz, DMSO-d_6)

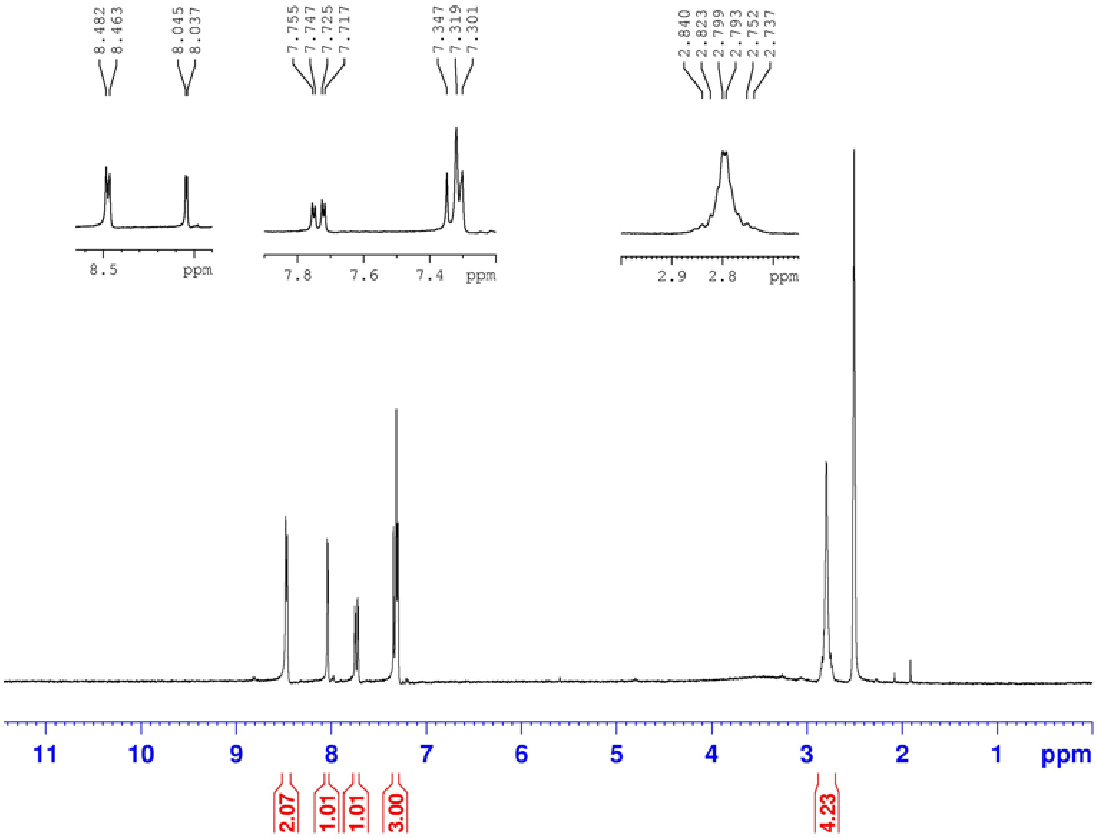

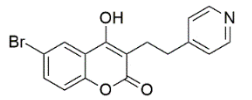

11

^{13}C NMR (300 MHz, DMSO-d$_6$)

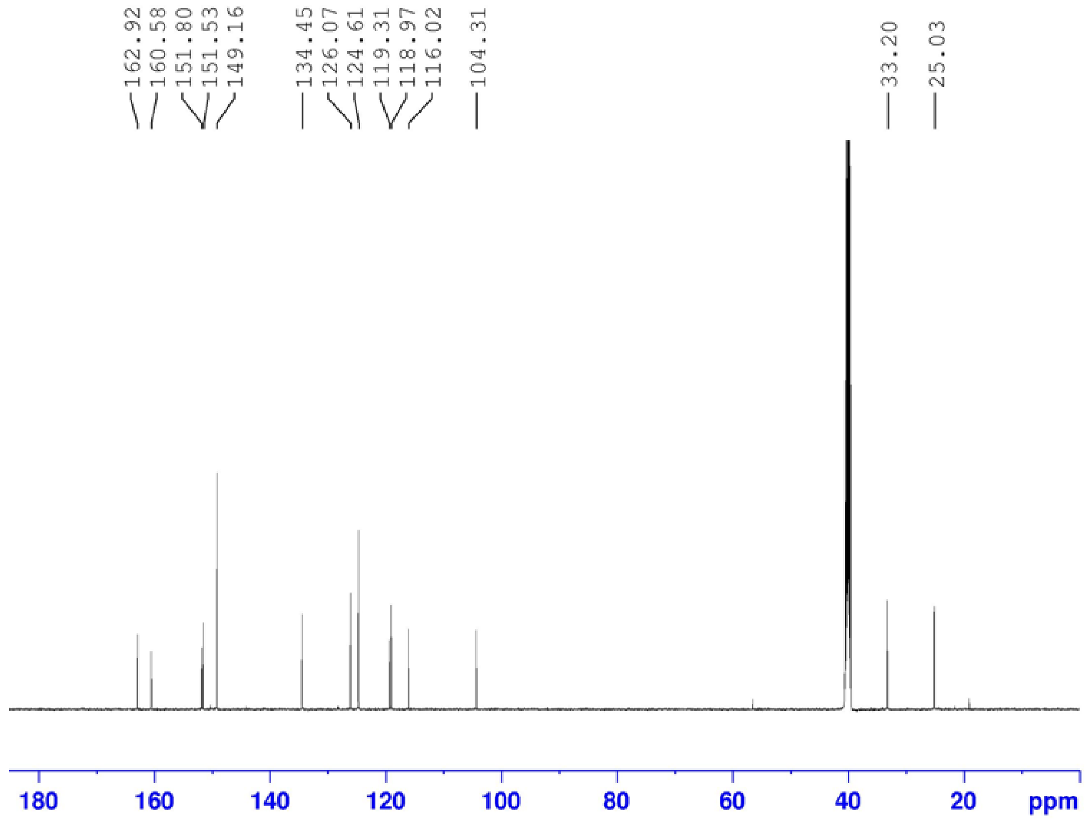

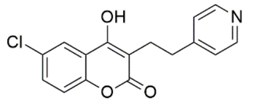

12

^{1}H NMR (500 MHz, d_6-DMSO)

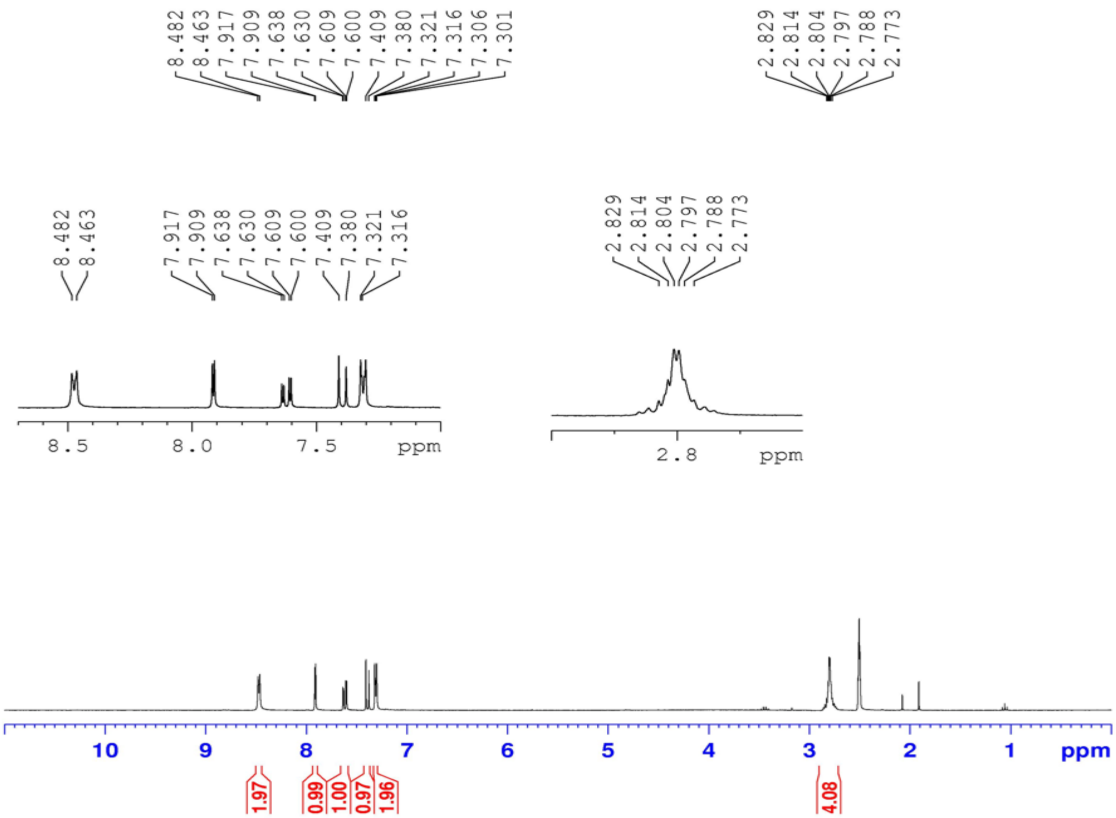

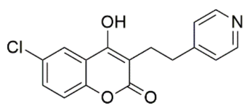

12

^{13}C NMR (500 MHz, d_6-DMSO)

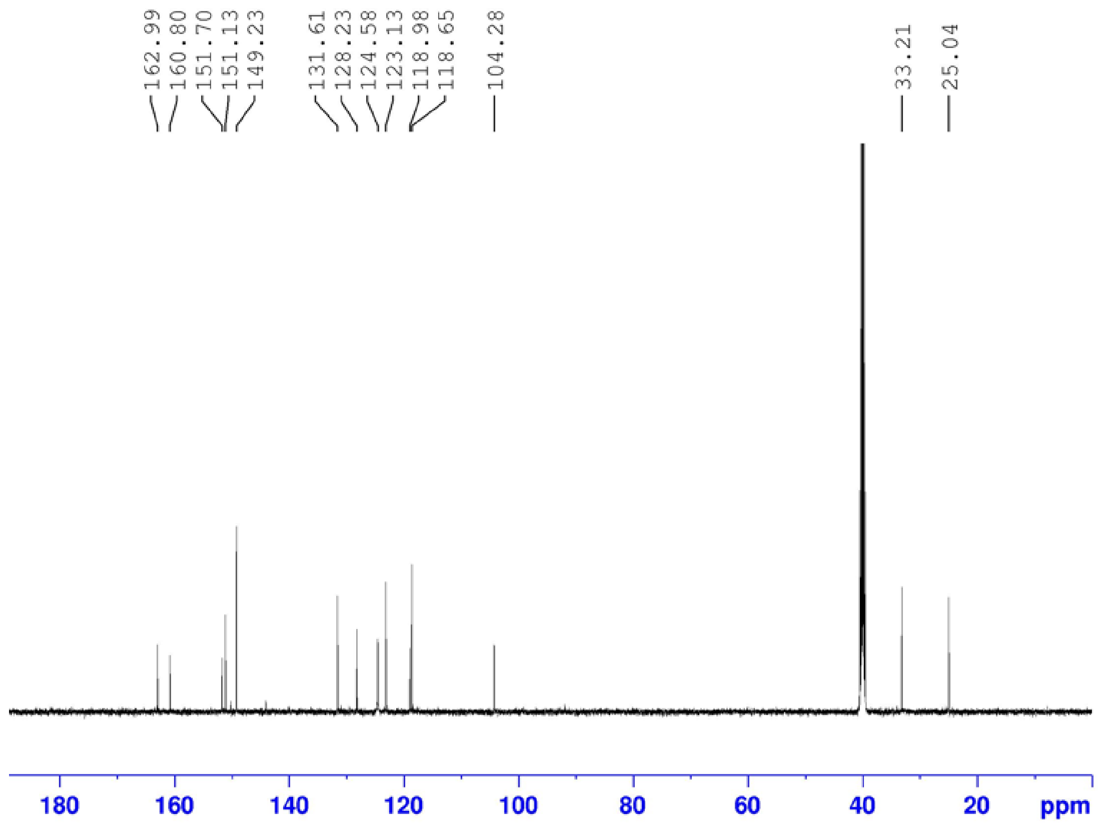

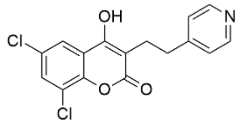

13

^{1}H NMR (500 MHz, DMSO-d_6)

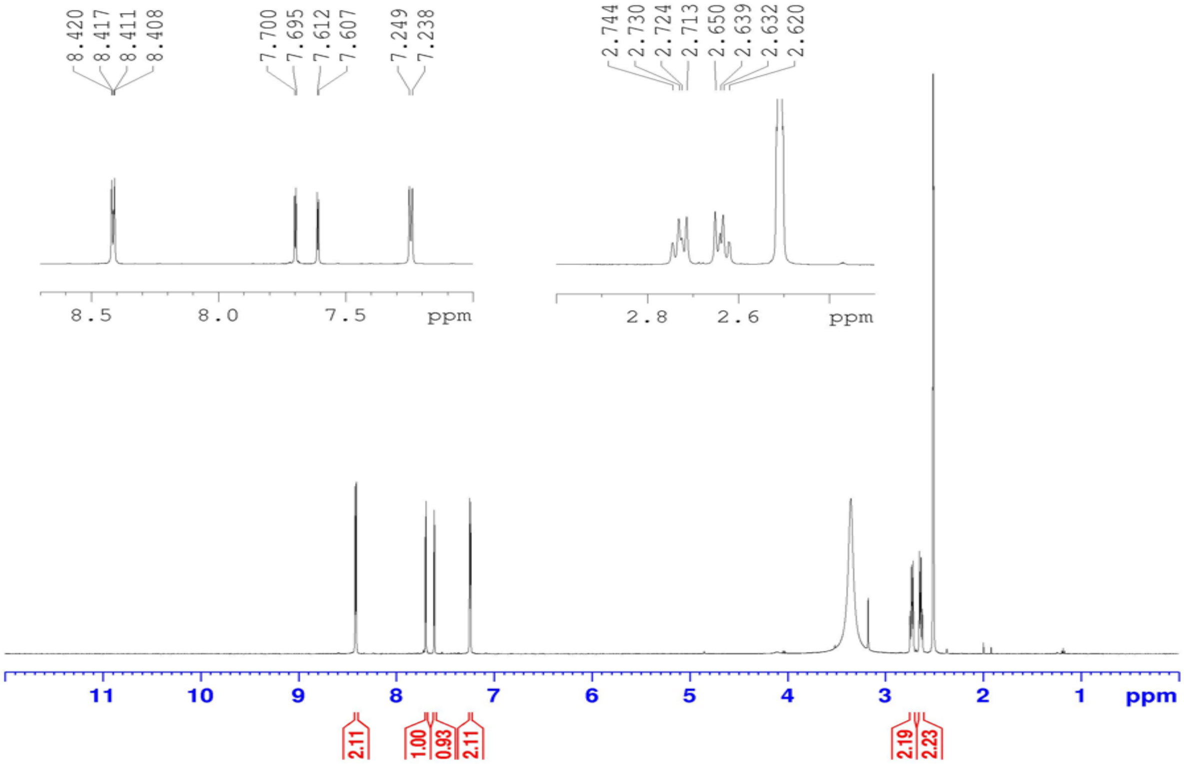

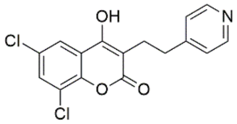

13

^{13}C NMR (500 MHz, DMSO-d_6)

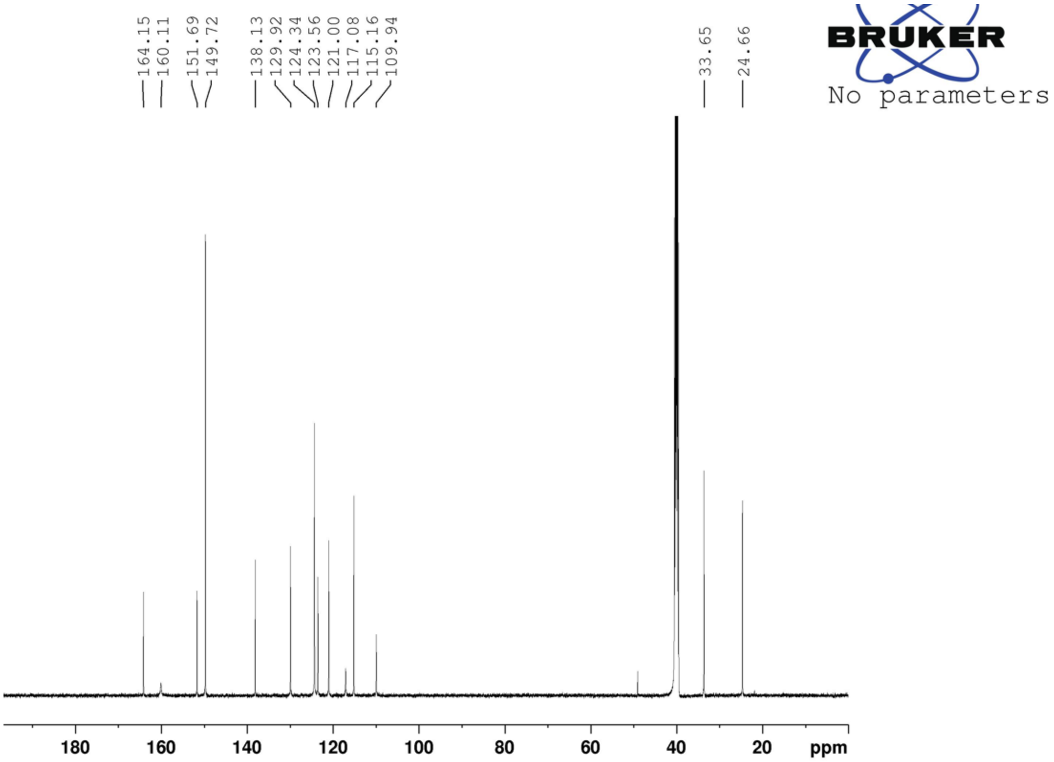

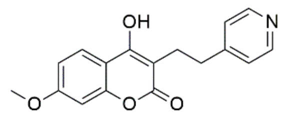

14

^{1}H NMR (300 MHz, DMSO-d_6)

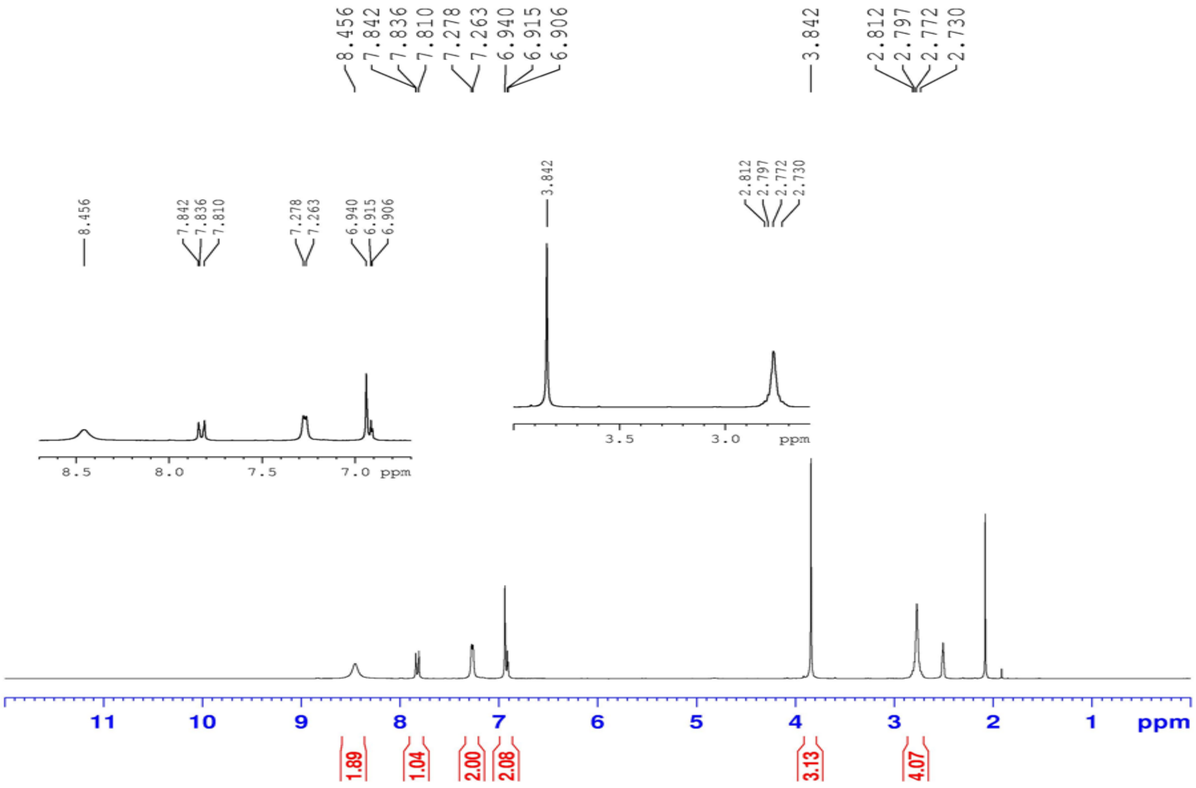

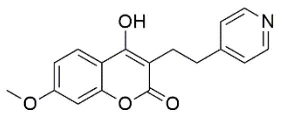

14

^{13}C NMR (300 MHz, DMSO-d_6) spectra of **14.**

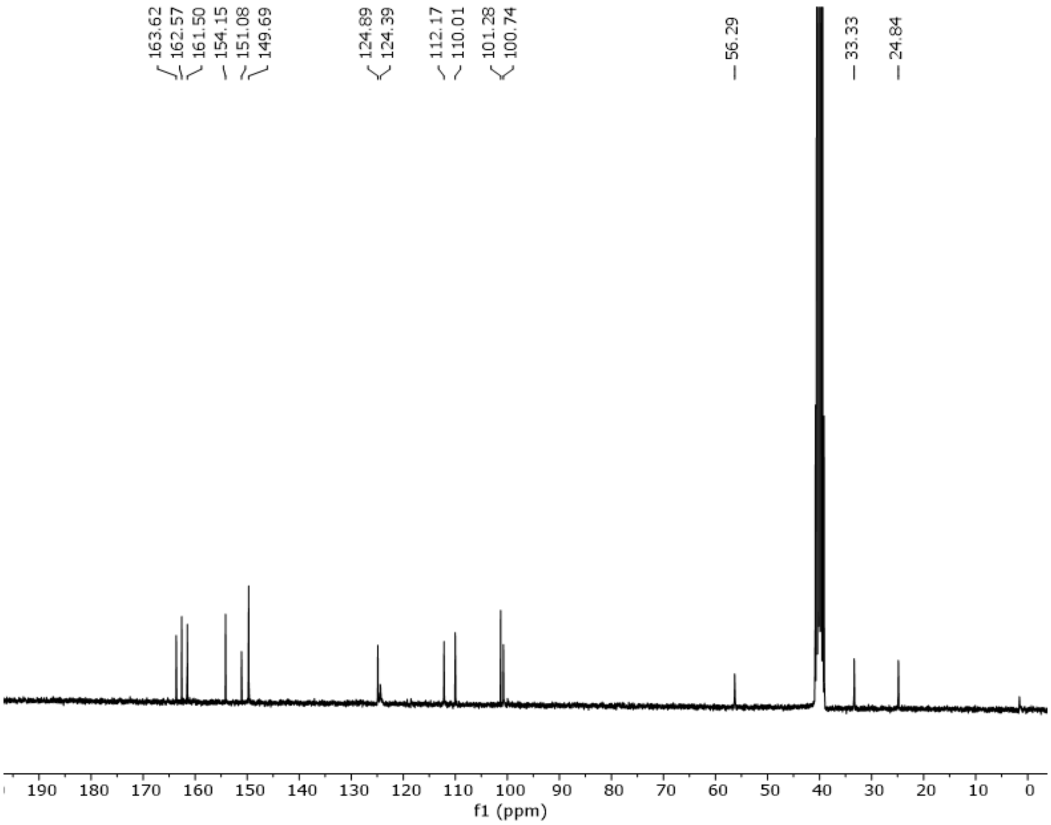

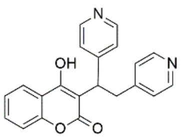

16

^{1}H NMR (500 MHz, d_4-methanol)

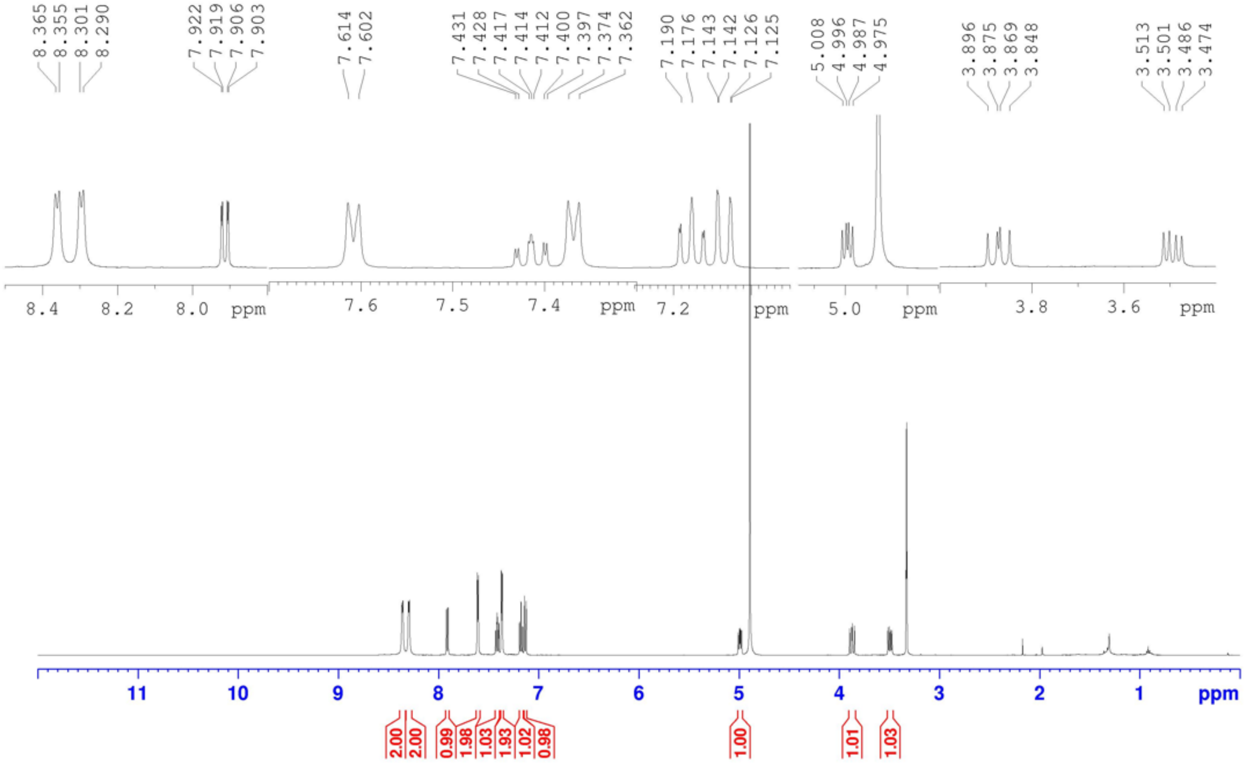

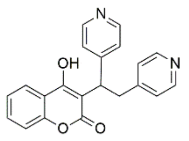

16

^{13}C NMR (500 MHz, d_4-methanol)

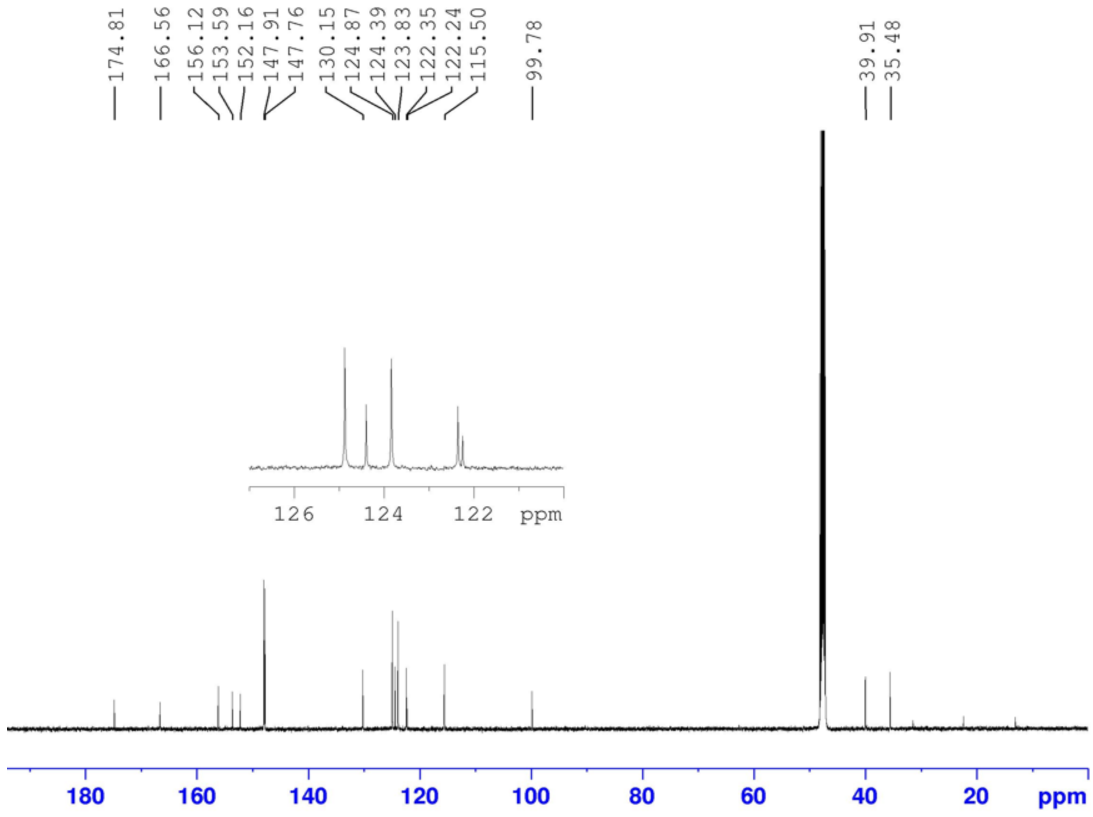

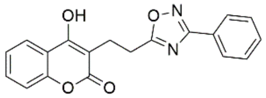

18

^{1}H NMR (500 MHz, d_6-DMSO)

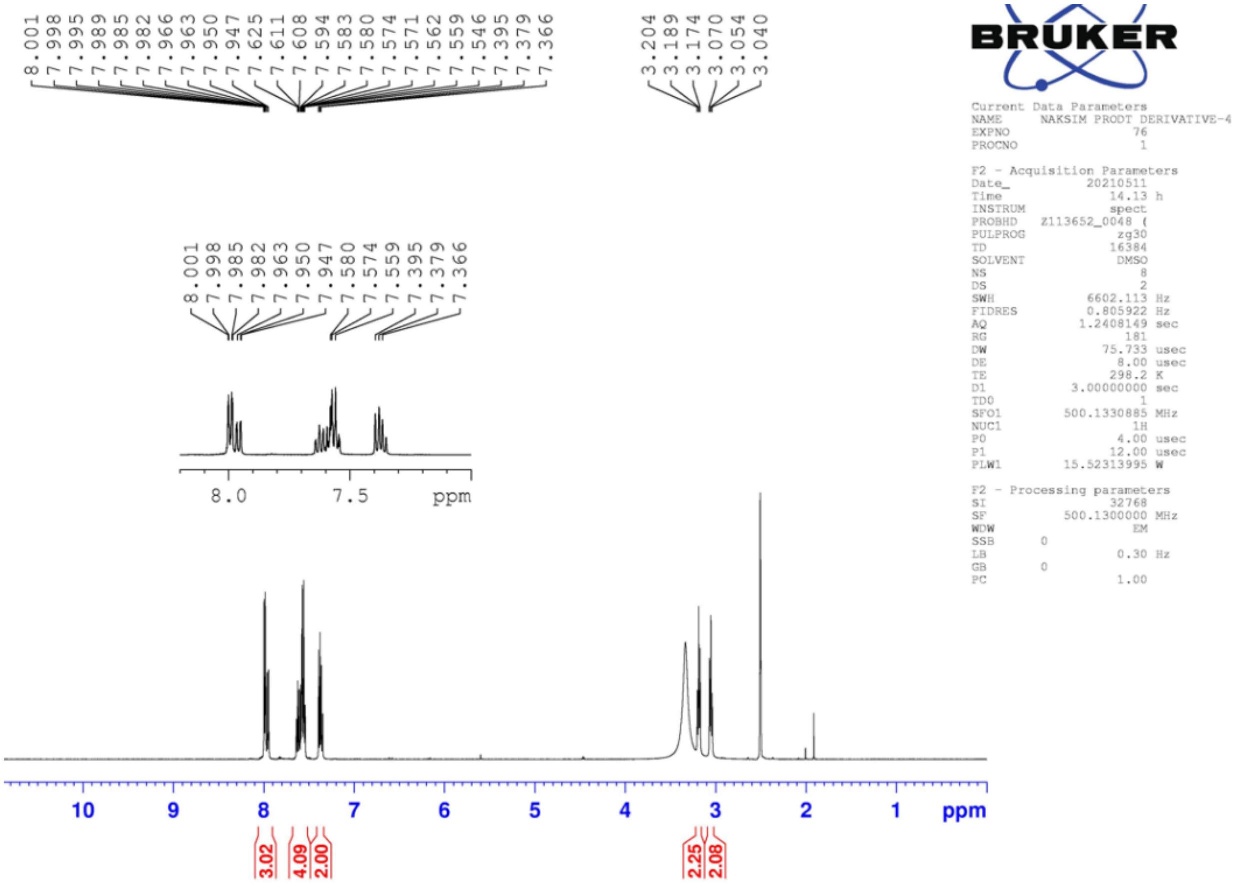

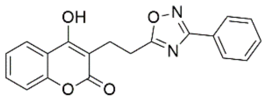

18

^{13}C NMR (500 MHz, d_6-DMSO)

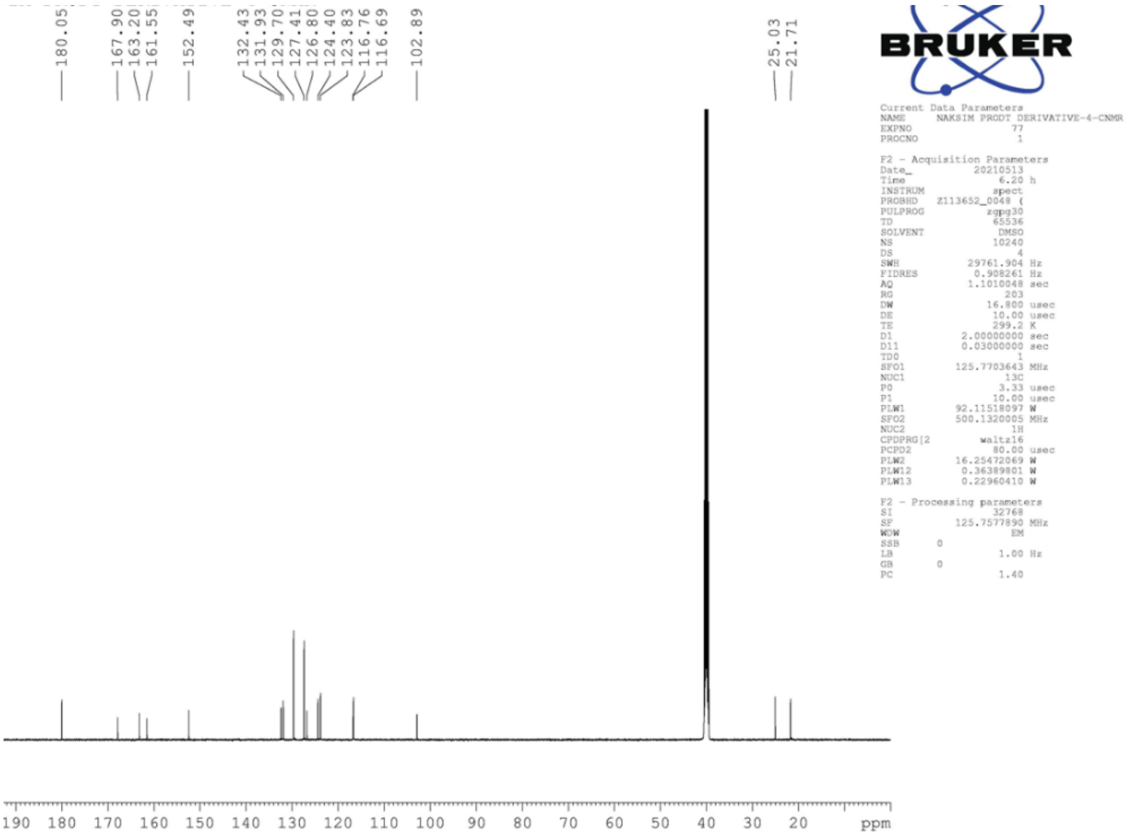

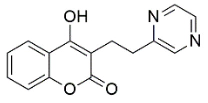

19

^{1}H NMR (500 MHz, d_6-DMSO)

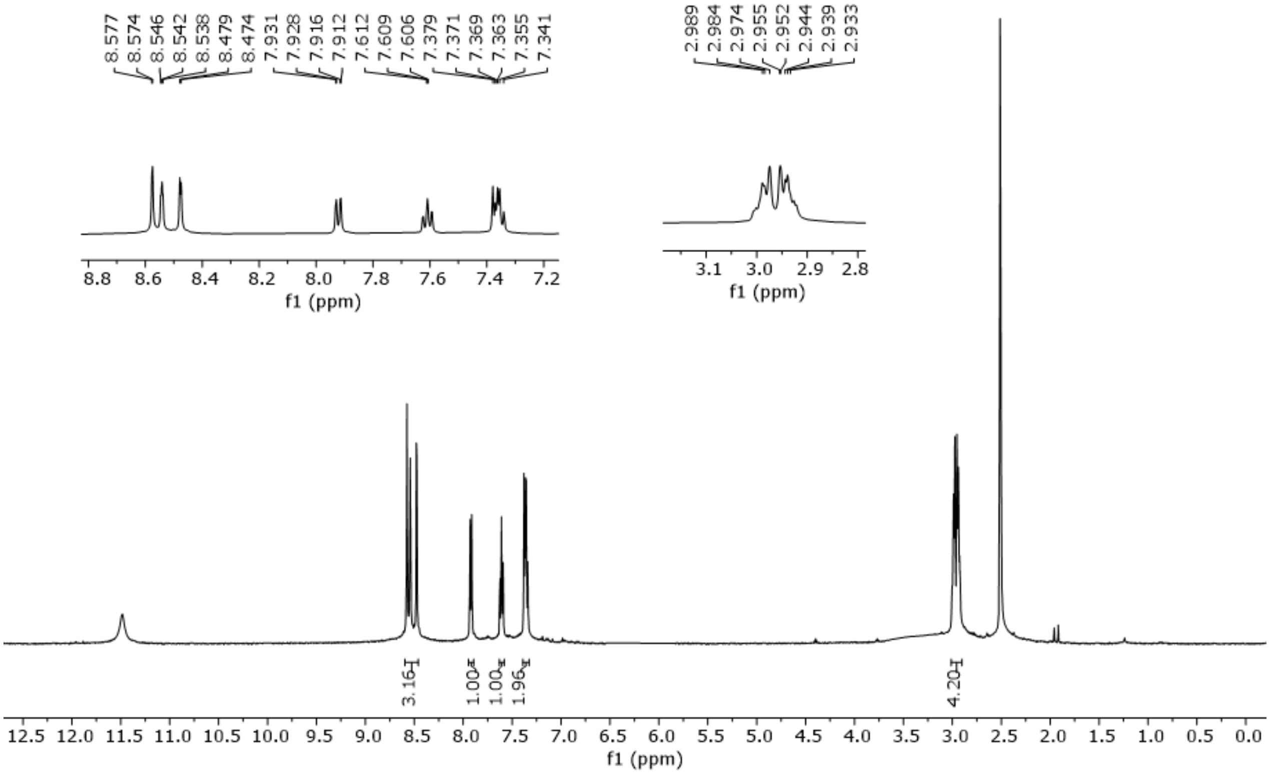

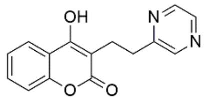

19

^{13}C NMR (500 MHz, d_6-DMSO)

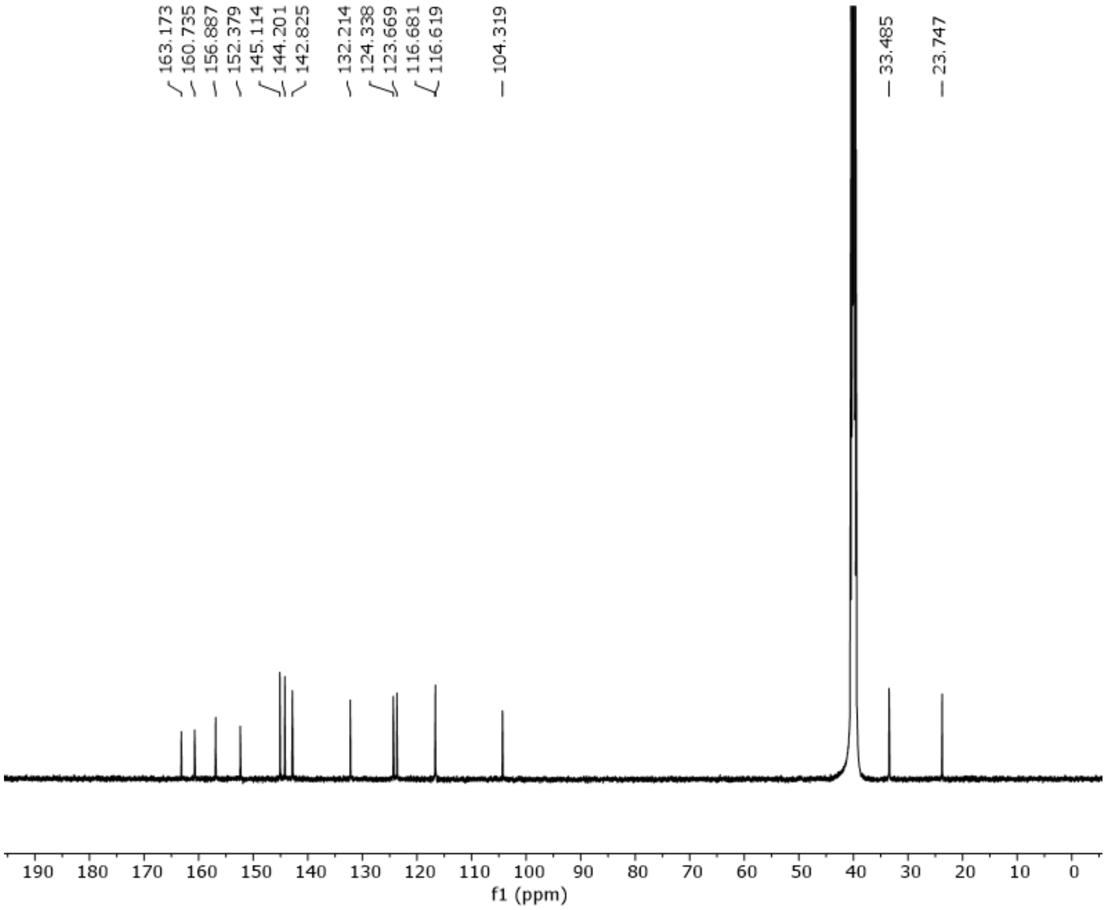

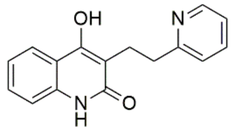

20

^{1}H NMR (500 MHz, d_6-DMSO)

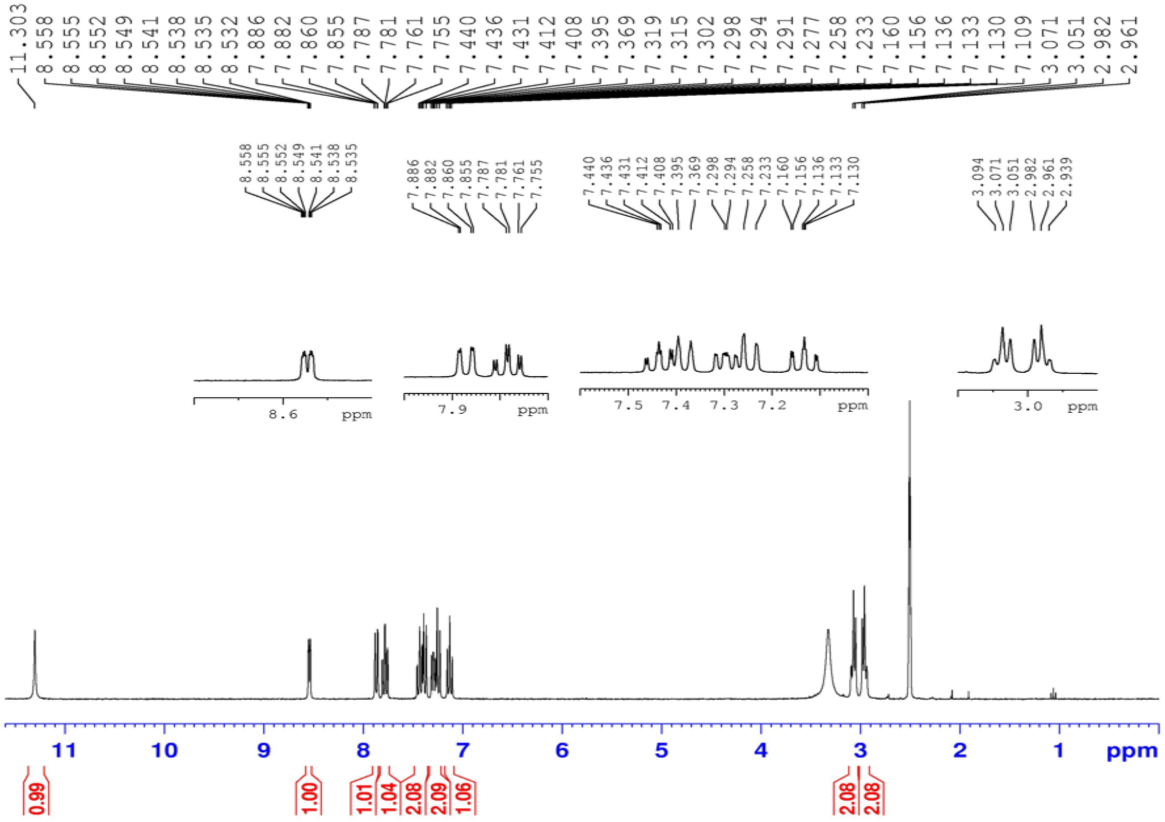

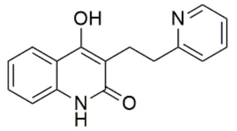

20

^{13}C NMR (500 MHz, d_6-DMSO)

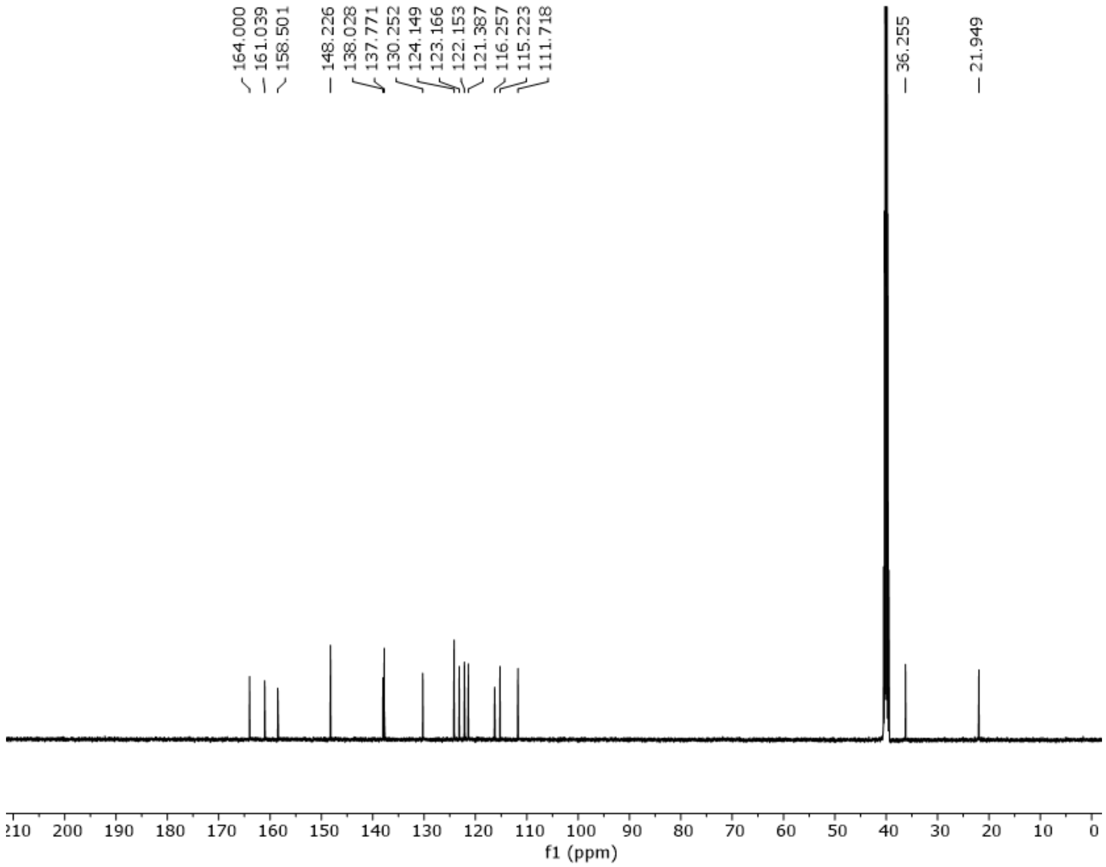

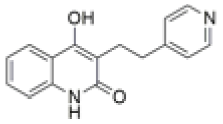

21

^{1}H NMR (300 MHz, d_6-DMSO)

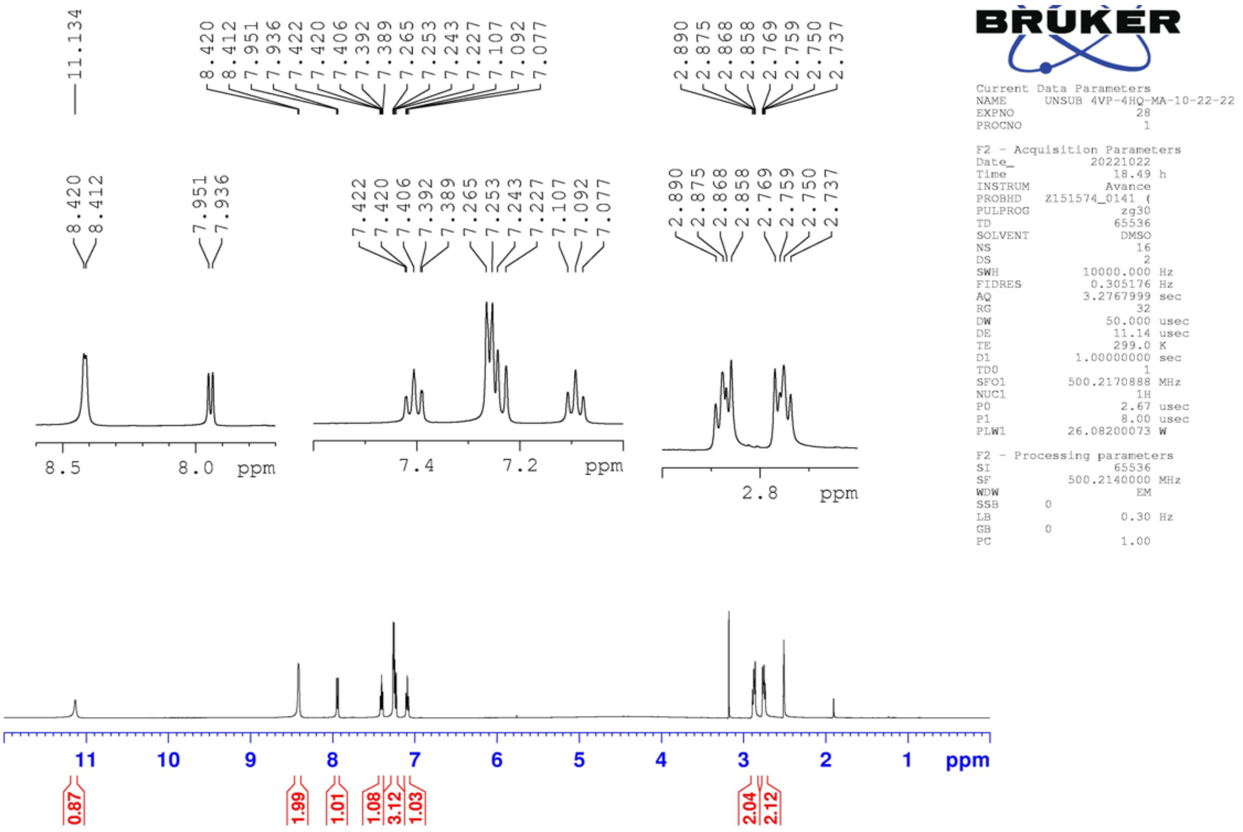

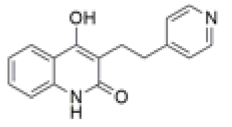

21

^{13}C NMR (300 MHz, d_6-DMSO)

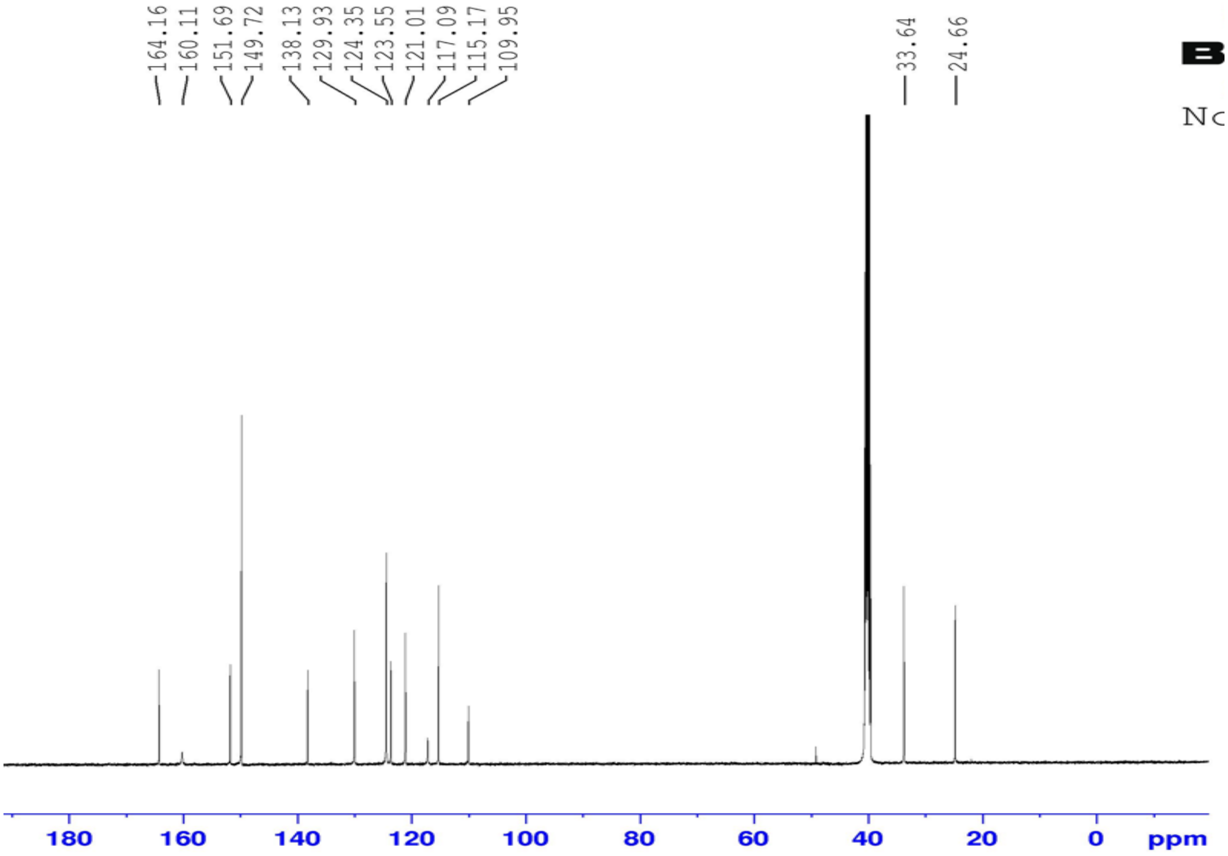

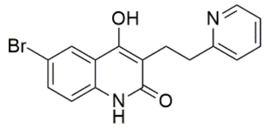

22

^{1}H NMR (300 MHz, d_6-DMSO)

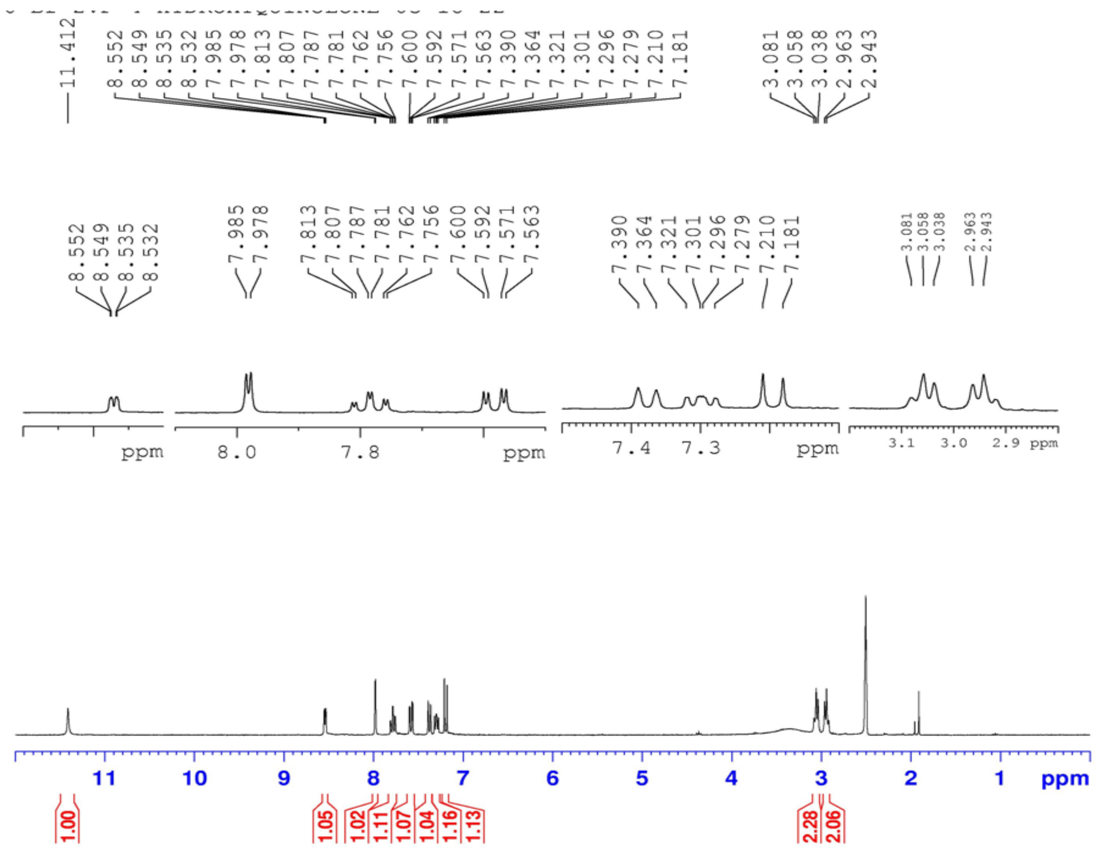

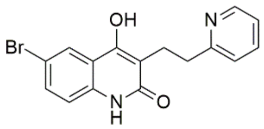

22

^{13}C NMR (300 MHz, d_6-DMSO)

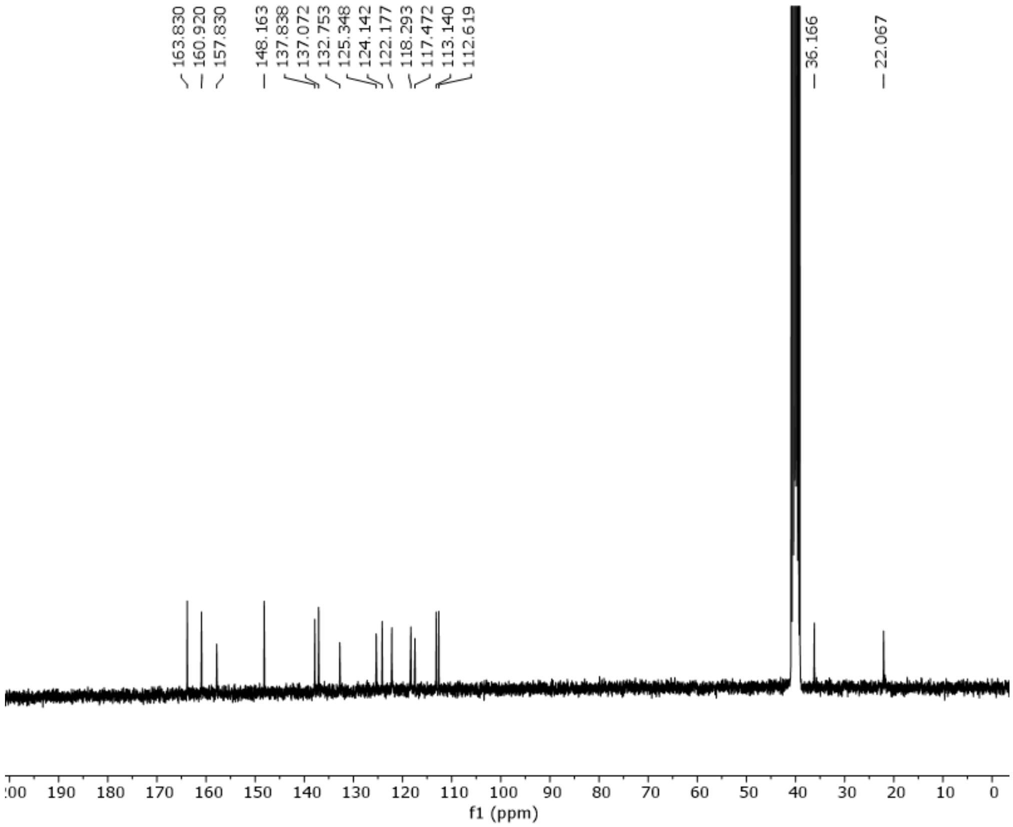

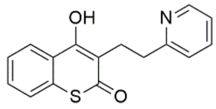

23

^{1}H NMR (500 MHz, d_6-DMSO)

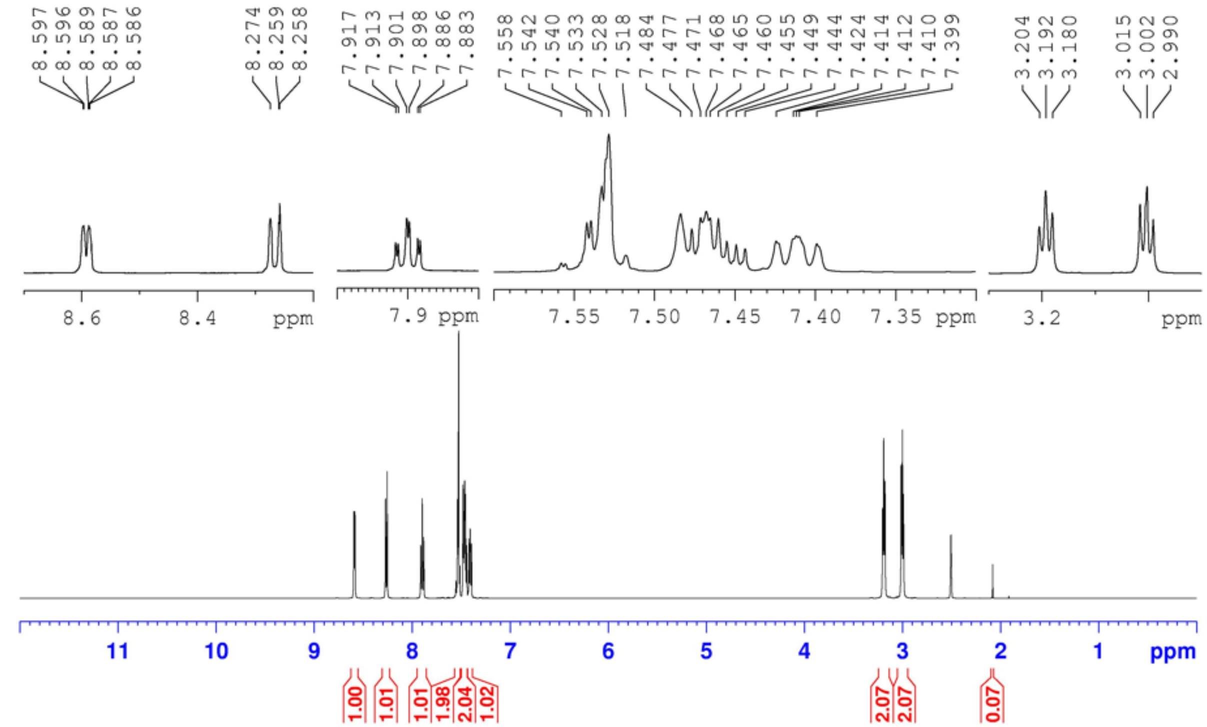

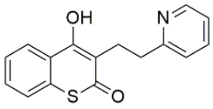

23

^{13}C NMR (300 MHz, d6-DMSO)

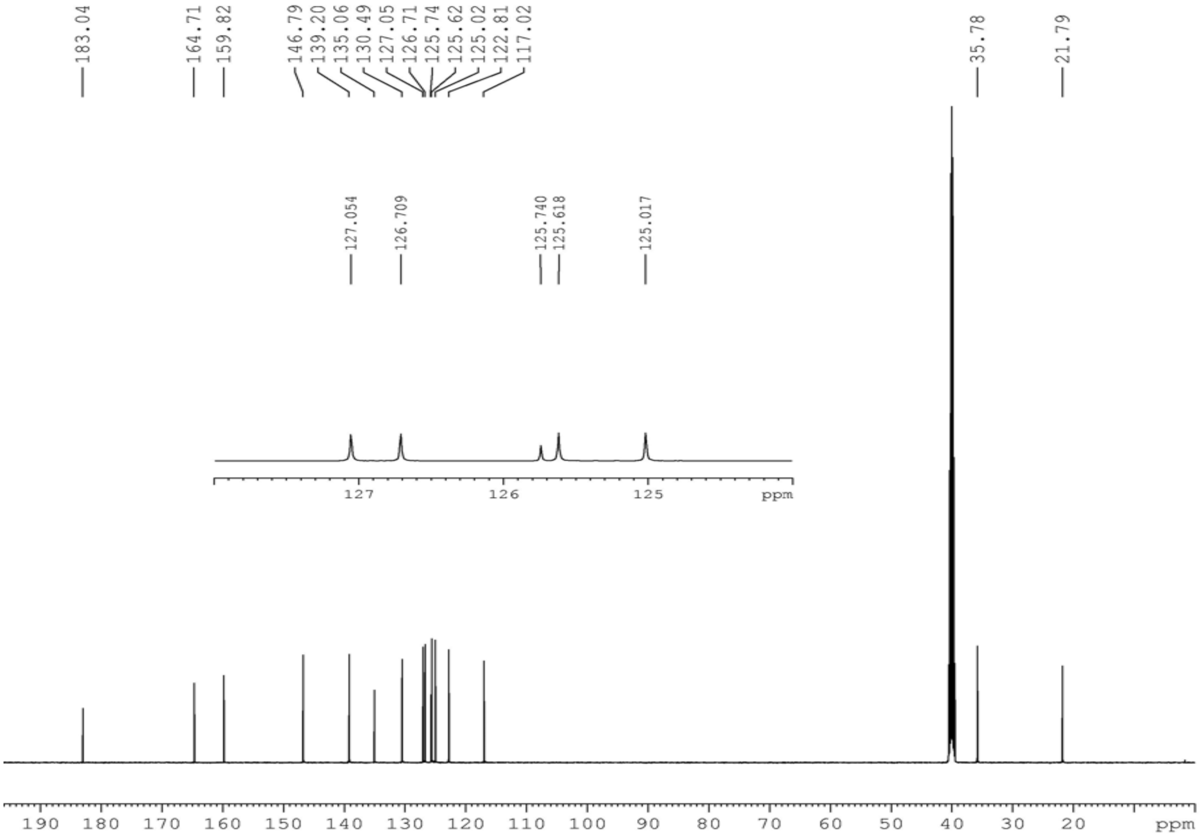

Supplement: RA-013-D3RA00251A-s001 [file RA-013-D3RA00251A-s001.pdf]
